# Long-term healthcare utilization and costs of babies born after assisted reproductive technologies (ART): a record linkage study with 10-years’ follow-up in England

**DOI:** 10.1093/humrep/dead198

**Published:** 2023-10-07

**Authors:** Xinyang Hua, Oliver Rivero-Arias, Maria A Quigley, Jennifer J Kurinczuk, Claire Carson

**Affiliations:** National Perinatal Epidemiology Unit, Nuffield Department of Population Health, University of Oxford, Oxford, UK; Centre for Health Policy, Melbourne School of Population and Global Health, The University of Melbourne, Parkville, VIC, Australia; National Perinatal Epidemiology Unit, Nuffield Department of Population Health, University of Oxford, Oxford, UK; National Perinatal Epidemiology Unit, Nuffield Department of Population Health, University of Oxford, Oxford, UK; National Perinatal Epidemiology Unit, Nuffield Department of Population Health, University of Oxford, Oxford, UK; National Perinatal Epidemiology Unit, Nuffield Department of Population Health, University of Oxford, Oxford, UK

**Keywords:** assisted reproduction, fertility treatment, healthcare cost, IVF/ICSI outcome, longitudinal follow-up

## Abstract

**STUDY QUESTION:**

Is the long-term health care utilization of children born after ART more costly to the healthcare system in England than children born to mothers with no fertility problems?

**SUMMARY ANSWER:**

Children born after ART had significantly more general practitioner (GP) consultations and higher primary care costs up to 10 years after birth, and significantly higher hospital admission costs in the first year after birth, compared to children born to mothers with no fertility problems.

**WHAT IS KNOWN ALREADY:**

There is evidence that children born after ART are at an increased risk of adverse birth outcomes and a small increased risk of rare adverse outcomes in childhood.

**STUDY DESIGN, SIZE, DURATION:**

We conducted a longitudinal study of 368 088 mother and baby pairs in England using a bespoke linked dataset. Singleton babies born 1997–2018, and their mothers, who were registered at GP practices in England contributing data to the Clinical Practice Research Datalink (CPRD), were identified through the CPRD GOLD mother–baby dataset; this data was augmented with further linkage to the mothers’ Human Fertilisation and Embryology Authority (HFEA) Register data. Four groups of babies were identified through the mothers’ records: a ‘fertile’ comparison group, an ‘untreated sub-fertile’ group, an ‘ovulation induction’ group, and an ART group. Babies were followed-up from birth to 28 February 2021, unless censored due to loss to follow-up (e.g. leaving GP practice, emigration) or death.

**PARTICIPANTS/MATERIALS, SETTING, METHODS:**

The CPRD collects anonymized coded patient electronic health records from a network of GPs in the UK. We estimated primary care costs and hospital admission costs for babies in the four fertility groups using the CPRD GOLD data and the linked Hospital Episode Statistics (HES) Admitted Patient Care (APC) data. Linear regression was used to compare the care costs in the different groups. Inverse probability weights were generated and applied to adjust for potential bias caused by attrition due to loss to follow-up.

**MAIN RESULTS AND THE ROLE OF CHANCE:**

Children born to mothers with no fertility problems had significantly fewer consultations and lower primary care costs compared to the other groups throughout the 10-years’ follow up. Regarding hospital costs, children born after ART had significantly higher hospital admission costs in the first year after birth compared to those born to mothers with no fertility problems (difference = £307 (95% CI: 153, 477)). The same pattern was observed in children born after untreated subfertility and ovulation induction.

**LIMITATIONS, REASONS FOR CAUTION:**

HFEA linkage uses non-donor data cycles only, and the introduction of consent for data use reduced the availability of HFEA records after 2009. The fertility groups were derived by augmenting HFEA data with evidence from primary care records; however, there remains some potential misclassification of exposure groups. The cost of neonatal critical care is not captured in the HES APC data, which may cause underestimation of the cost differences between the comparison group and the infertility groups.

**WIDER IMPLICATIONS OF THE FINDINGS:**

The findings can help anticipate the financial impact on the healthcare system associated with subfertility and ART, particularly as the demand for these treatments grows.

**STUDY FUNDING/COMPETING INTEREST(S):**

C.C. and this work were funded by a UK Medical Research Council Career Development Award [MR/L019671/1] and a UK MRC Transition Support Award [MR/W029286/1]. X.H. is an Australia National Health and Medical Research Council (NHMRC) Emerging Leadership Fellow [grant number 2009253]. The authors declare no competing interest.

**TRIAL REGISTRATION NUMBER:**

N/A.

## Introduction

One in six couples worldwide experience some form of fertility problem during their reproductive lifetime (ESHRE, 2020). The use of ART in helping couples with fertility problems to conceive continues to increase. Since the first successful *in vitro* fertilization (IVF) pregnancy in 1978, over 10 million children have been born worldwide using these techniques (ESHRE, 2020). Both treatment rates and success rates have been rising in the recent decades; in the UK, the number of embryos transferred increased from 12 288 in 1991 to 70 465 by 2008, with IVF success rates being tripled in all age bands (HFEA, 2020).

With the growing number of babies born after ART, it is important to understand their longer-term health outcomes, and to consider whether their treatment pathways are associated with additional cost implications for the healthcare system. While most ART births result in healthy children, there is evidence that they are at an increased risk of adverse birth outcomes which result in longer hospital stays in early life (e.g. preterm birth, delivery by caesarean section) and are only partly due to multiple pregnancy (Helmerhorst *et al.*, 2004; Declercq *et al.*, 2015). There is evidence of rare adverse outcomes among these children, including an increase in congenital malformations (Hansen *et al.*, 2013) and imprinting disorders (Cortessis *et al.*, 2018). Epidemiological research examining the risks of neurodevelopmental conditions (Sandin *et al.*, 2013), growth (Bay *et al.*, 2019), cardiometabolic effects (Guo *et al.*, 2017), and asthma (Carson *et al.*, 2013) have tended to suggest a small increased risk for this group.

There is limited understanding about whether the reported adverse birth and child outcomes associated with ART will translate into higher healthcare utilization and costs in the longer term. This information is important to help anticipate the financial impact on the healthcare system and inform resource allocation and policies. Previous studies looking at costs associated with ART have mainly focus on the treatment cost itself (Chambers *et al.*, 2009; Bahadur *et al.*, 2020), or the neonatal period and birth-admission only, with the cost mainly driven by multiple birth and low birth weight (Koivurova *et al.*, 2004; Ledger *et al.*, 2006; Chambers *et al.*, 2007). Very few studies have investigated longer-term health care utilization and costs associated with ART (Koivurova *et al.*, 2007; Dukhovny *et al.*, 2021). None of these previous studies has incorporated primary health care usage and costs into their evaluation.

The aim of this study was to identify any additional primary health care and hospital admission costs associated with the longer-term care of children born to mothers with fertility problems and born through ART in England.

## Materials and methods

### Data source

The PEARL (Prolonged Effects of Assisted reproductive technologies on women and children’s health: a Record Linkage study for England) study is designed to assess the impact of successful fertility treatment on the long-term health of women and their children in England. To do this, a new bespoke linked dataset was created to link information on fertility treatment held in the Human Fertility and Embryology Authority (HFEA) Register to health data held in the Clinical Practice Research Datalink (CPRD GOLD, which comprises primary care data, and is linked to the Hospital Episode Statistics (HES) and Index of Multiple Deprivation). The CPRD GOLD collects anonymized fully coded patient electronic health records from a network of general practitioners (GPs) across over 600 primary care practices in the UK, capturing information on demographic characteristics, diagnoses and symptoms, referrals to hospital and specialist care, tests and prescriptions issued (Herrett *et al.*, 2015). CPRD includes a practice-specific family number that can be used to identify people within the same family, allowing a link between mothers with their children registered at the same practice. CPRD provides routine data linkages between primary care and other datasets, including to the HES Admitted Patient Care (HES APC) data, and the Index of Multiple Deprivation (IMD). Linked HES APC contains the hospital episode information (admission and discharge dates, diagnoses, procedures, etc.) of admissions to English NHS healthcare providers from April 1997 (Herbert *et al.*, 2017), however, granular detail on neonatal and paediatric critical care are not available. Since 1991, UK legislation has required details of all fertility treatment cycles to be recorded in the HFEA register. Prior to 2009 couples were not asked for consent for data to be used for research, but these data can be used with appropriate permissions. After the introduction of consent for data use, only data from couples who gave consent can be included. In addition, the law prohibits the sharing of data on donor cycles. The available details of non-donor fertility cycles recorded in the HFEA were linked to the CPRD dataset in a bespoke linkage process; details of the linkage process and the legal basis are provided in the Supplementary Materials and Methods and Supplementary Figs S1 and S2.

### Study sample

Babies born 1991–2009, and their mothers, who were registered at CPRD-contributing GP practices in England, were identified through the CPRD GOLD mother–baby dataset. Women and their children flagged as mother–baby pairs in the linked dataset were eligible for inclusion if the mothers had been registered for 18 months prior to the birth of the child (to allow identification of fertility history) and had given consent for linkage to HES and other data sources. Babies were followed up from birth to 20 February 2021, unless censored due to loss to follow up (e.g. emigration, death or, for primary care only, leaving GP practice). To allow comparison of primary care and hospitalization costs, we have restricted the main analysis to singletons, born 1997–2017, with available HES linkage. A flow diagram describing the derivation of the study population is shown in Supplementary Fig. S3.

### Conception history and other variables

Mother–baby pairs were grouped based on the evidence of conception history in the mother’s data: a ‘fertile’ comparison group (no evidence of consultations, investigations, or treatment for fertility problems in the primary care record), an ‘untreated sub-fertile’ group (evidence of consulting GP for concerns about time taken to conceive or diagnosis of fertility problem/past treatment, and a conception with no further evidence of treatment), an ‘ovulation induction’ group (evidence of ovulation induction medication, such as clomiphene citrate, from notes or product codes of the prescription), and an ART group (ART indicated for this pregnancy by HFEA linkage, augmented with records in mother’s GP notes of ART including IVF or ICSI, relevant prescriptions or of referral for ART). Details of the codes used to identify these groups in primary care records, which were developed with specialist clinical input, are provided in the Supplementary Materials and Methods.

The maternal characteristics and health behaviours, and the baby’s characteristics, for the population were described. Maternal age at birth was derived from mother and baby year of birth and grouped into 5-year bands (<25, 25–29, 30–34, 35–39, ≥40 years). Maternal ethnicity (white/minority ethnic group), smoking history (ever/never), and body mass index (BMI, kg/m^2^) prior to pregnancy were derived based on existing code lists (Springate *et al.*, 2014). The baby’s sex and year of birth were drawn from the CPRD record, and evidence of low birthweight or preterm birth (yes/no) or multiple birth (yes/no) were generated based on mother and baby primary care records, administrative details when registered to the practice, and HES records, where available. Deprivation was measured by the linked IMD (quintiles), at the individual patient level and GP practice level.

### Estimation of costs

Primary care costs were calculated as the sum of the cost for consultations, tests, referrals for outpatient hospital care, and prescriptions. Resources used in each of these categories were extracted from the CPRD data. Consultations were grouped by type (e.g. face-to-face surgery, visit, telephone, etc.) and staff role (e.g. GP, nurse, etc.) and attached to corresponding unit costs collected from the Unit Costs of Health and Social Care report (Curtis and Burns, 2015). Tests were grouped into broad categories (e.g. clinical biochemistry, immunology, scans, etc.) and attached to corresponding unit costs collected from NHS reference costs (NHS, 2019). Referral records from the CPRD were used as a proxy of patients’ first visit to outpatient clinics and were attached to NHS reference costs based on specialty. A British National Formulary (BNF) code was available for each prescription record in the CPRD data, which was attached to the unit cost obtained from the Prescription Cost Analysis England. More details on the costing methods for primary care resources use can be found in Supplementary Materials and Methods.

Hospital admission costs were estimated using the HES APC data. In HES APC, each data record indicates a Finished Consultant Episode (FCE), which represents a continuous period of care under one consultant. As suggested by the Department of Health, costs were estimated at the episode level. The 2017-18 Casemix Grouper Software (HRG4+) was used to help allocate each FCE to a Healthcare Resource Group (HRG) (National-Casemix-Office, 2018), primarily based on any procedures carried out, diagnoses, hospital admission type, episode length of stay, and patient characteristics. HRGs are standard groupings of clinically similar treatments which use comparable levels of healthcare resources. The NHS reference cost schedules were used to price the HRGs (NHS, 2019).

All costs were inflated to 2018–2019 prices with the New Health Services Index using the consumer prices index (CPI) (Health) published in the 2019 Unit Costs of Health and Social Care report (Curtis and Burns, 2019).

### Statistical methods

Baseline characteristics were described and compared across fertility groups; for categorical variables, frequency and proportions are presented with a *P*-value for Pearson chi-squared test, and for continuous variables, a mean and SD are presented with a *P*-value for ANOVA. Based on dates of the services or admissions, the number of consultations and associated costs were aggregated into half-year intervals. The number of consultations, primary care costs, and hospital admission costs from birth to up to 10 years were identified according to the four different fertility groups.

Linear regression was conducted in each time (half-year) interval to compare the costs in the different groups, with clustering by mother’s ID to account for within family similarities. Inverse probability weights (IPW) were generated using a Cox model and applied in the linear regressions to adjust for potential bias caused by attrition (mainly due to patients leaving the contributing GP practice) from the CPRD primary care data (Hernán *et al.*, 2004) (see Supplementary Materials and Methods). No further adjustment based on baseline characteristics was conducted, as we were interested in the differences in total costs between conception groups in a real world scenario. The costs were aggregated into 1st, 2nd, 3rd–5th, and 6th–10th years’ costs and compared across the different fertility groups, and 95% confidence intervals (95% CIs) around the aggregated costs at each time point were derived using non-parametric bootstrapping with 1000 replications. Forest plots were used to compare the differences in aggregated costs between the comparison group and the untreated sub-fertile and fertility treatment groups.

### Additional analyses

The study population presented here includes singletons only, with births limited to 1997 onwards to allow linkage to HES (both mother’s data for birth details and baby’s data for hospital admissions). The analyses outlined above were first repeated in twins/higher order multiples only, born 1997–2017, with HES linkage. Then, primary care utilization and costs were analyzed in all babies in the primary care dataset born 1992–2017 without restricting to those with linked HES data.

### Ethical approval

The finding reported here is part of the PEARL study (Prolonged Effects of ART: a Record Linkage study for England). Two related datasets were used. Preliminary work was conducted using CPRD data with linkage to HES only. This first stage was approved by the CPRD Research Data Governance (RDG) process (ref: 15_090R), and is covered by CPRD’s overarching Confidentiality Advisory Group (CAG) approval from the Health Research Authority for the use of anonymized patient data in research. The analysis and results presented here were built on that preliminary work, using a bespoke linked dataset that adds fertility records from the HFEA register. The second stage was approved by CPRD’s RDG process (ref: 16_215R), South Central Hampshire—B Research Ethics Committee (16/SC/0222), CAG (16/CAG/0053), and HFEA Research Register Panel (HFEARRPCarson01-01). The legal basis for the transfer, processing, and linkage of the bespoke dataset is included in Supplementary Material S1.

## Results

In total, 368 088 singleton babies born between 1997 and 2017 were included in the main analysis (a population flow chart is shown in Supplementary Fig. S3, detailing the main and additional analysis samples). Statistics describing follow up of the study cohort can be found in Supplementary Table S1. Complete 10-year follow-up was available for 97 168 children (26.4%) at the analytical end date of 28 February 2021. Baseline characteristics of mothers and babies included in this study are presented in Table 1. Among the 368 088 singleton babies included in the study, 341 863 (92%) were born without any maternal record of fertility problems, 19 333 (5.3%) were born following untreated sub-fertility, 2291 (0.6%) were born following ovulation induction, and 4601 (1.2%) were born through ART. Compared to mothers who conceived without fertility problems, mothers with untreated subfertility and those who conceived through ovulation induction or ART were older when they had their children. A greater proportion of those conceived after ART were in the least deprived quintile of IMD, and were more likely to be of low birthweight and/or born preterm, compared to other groups.

**Table 1.** Characteristics of singleton mother–baby pairs, by conception history group.^a^

	All singletons	No fertility problems	Untreated subfertility	Ovulation induction	ART	*P*-value (all exposure groups)	***P*-value (subfertile groups only)** ^b^
N (% of total)	368 088 (100%)	341 863 (92.3%)	19 333 (5.3%)	2291 (0.6%)	4601 (1.2%)		
**Mum’s characteristics**							
**Age at delivery**						<0.001	<0.001
<25	66 496 (18.1%)	64 874 (19.0%)	1448 (7.5%)	125 (5.5%)	49 (1.1%)		
25–29	90 435 (24.6%)	85 623 (25.0%)	3846 (19.9%)	542 (23.7%)	424 (9.2%)		
30–34	118 299 (32.1%)	109 121 (31.9%)	6842 (35.4%)	904 (39.5%)	1432 (31.1%)		
35–39	74 222 (20.2%)	66 399 (19.4%)	5370 (27.8%)	548 (23.9%)	1905 (41.4%)		
≥40	18 636 (5.1%)	15 846 (4.6%)	1827 (9.5%)	172 (7.5%)	791 (17.2%)		
*Missing, n (% of all)*	*0 (0%)*	*0 (0%)*	*0 (0%)*	*0 (0%)*	*0 (0%)*		
**Ethnicity**						<0.001	0.66
White	95 697 (47.9%)	88 830 (48.1%)	5152 (45.0%)	502 (43.9%)	1213 (45.5%)		
Minority Ethnic group	104 181 (52.1%)	95 800 (51.9%)	6285 (55.0%)	642 (56.1%)	1454 (54.5%)		
*Missing, n (% of all)*	*168 210 (45.7%)*	*157 233 (46.0%)*	*7896 (40.8%)*	*1147 (50.1%)*	*1934 (42.0%)*		
**Smoking history**						<0.001	<0.001
Current	56 322 (32.5%)	53 402 (33.1%)	2406 (25.2%)	216 (23.2%)	298 (16.7%)		
Ex	28 493 (16.4%)	26 021 (16.2%)	1861 (19.5%)	162 (17.4%)	449 (25.1%)		
Never	88 549 (51.1%)	81 677 (50.7%)	5275 (55.3%)	555 (59.5%)	1042 (58.2%)		
*Missing, n (% of all)*	*194 724 (52.9%)*	*180 763 (52.9%)*	*9791 (50.6%)*	*1358 (59.3%)*	*2812 (61.1%)*		
**BMI before pregnancy**							
Mean (SD)	25.7 (5.9)	25.7 (5.9)	25.9 (5.9)	27.3 (6.6)	25.0 (4.7)	<0.001	<0.001
*Missing, n (% of all)*	*104 836 (28.5%)*	*102 040 (29.8%)*	*1685 (8.7%)*	*617 (26.9%)*	*1050 (22.8%)*		
**Child’s characteristics**							
**Year of birth**						<0.001	<0.001
1997–2003	99 467 (27.0%)	93 559 (27.4%)	3933 (20.3%)	824 (36.0%)	1151 (25.0%)		
2004–2008	111 006 (30.2%)	103 226 (30.2%)	5794 (30.0%)	723 (31.6%)	1263 (27.5%)		
2009–2013	113 558 (30.9%)	104 730 (30.6%)	6759 (35.0%)	615 (26.8%)	1454 (31.6%)		
≥2014	44 057 (12.0%)	40 348 (11.8%)	2847 (14.7%)	129 (5.6%)	733 (15.9%)		
*Missing, n (% of all)*	*0 (0.0%)*	*0 (0.0%)*	*0 (0.0%)*	*0 (0.0%)*	*0 (0.0%)*		
**Sex**						0.99	0.78
Male	190 230 (51.7%)	176 630 (51.7%)	10 051 (52.0%)	1181 (51.5%)	2368 (51.5%)		
Female	177 858 (48.3%)	165 233 (48.3%)	9282 (48.0%)	1110 (48.5%)	2233 (48.5%)		
*Missing, n (% of all)*	*0 (0.0%)*	*0 (0.0%)*	*0 (0.0%)*	*0 (0.0%)*	*0 (0.0%)*		
**IMD (patient level)**						<0.001	<0.001
Least deprived 1	80 498 (21.9%)	72 976 (21.4%)	5327 (27.6%)	647 (28.3%)	1548 (33.7%)		
2	77 316 (21.0%)	71 077 (20.8%)	4445 (23.0%)	532 (23.2%)	1262 (27.4%)		
3	70 257 (19.1%)	65 311 (19.1%)	3653 (18.9%)	474 (20.7%)	819 (17.8%)		
4	76 723 (20.9%)	72 239 (21.1%)	3434 (17.8%)	404 (17.6%)	646 (14.0%)		
Most deprived 5	63 006 (17.1%)	59 989 (17.6%)	2461 (12.7%)	232 (10.1%)	324 (7.0%)		
*Missing, n (% of all)*	*288 (0.01)*	*271 (0.1%)*	*13 (0.1%)*	*2 (0.1%)*	*2 (0.0%)*		
**IMD (practice level)**						<0.001	<0.001
Least deprived 1	54 179 (14.7%)	49 262 (14.4%)	3592 (18.6%)	347 (15.1%)	978 (21.3%)		
2	78 122 (21.2%)	71 938 (21.0%)	4492 (23.2%)	474 (20.7%)	1218 (26.5%)		
3	72 844 (19.8%)	67 589 (19.8%)	3793 (19.6%)	580 (25.3%)	882 (19.2%)		
4	80 702 (21.9%)	75 597 (22.1%)	3846 (19.9%)	416 (18.2%)	843 (18.3%)		
Most deprived 5	82 223 (22.3%)	77 460 (22.7%)	3610 (18.7%)	474 (20.7%)	679 (14.8%)		
*Missing, n (% of all)*	*18 (0.0%)*	*17(0.0%)*	*0(0.0%)*	*0(0.0%)*	*1(0.0%)*		
**Low birthweight or preterm birth** ^c^						<0.001	<0.001
No	21 248 (7.0%)	19 474 (6.9%)	1270 (7.8%)	155 (8.5%)	349 (9.6%)		
Yes	*65 422 (17.8%)*	*61 023 (17.9%)*	*2955 (15.3%)*	*477 (20.8%)*	*967 (21.0%)*		
*Missing, n (% of all)*	21 248 (7.0%)	19 474 (6.9%)	1270 (7.8%)	155 (8.5%)	349 (9.6%)		

IMD, Index of Multiple Deprivation.

a Continuous variables are presented as mean (SD). Category variables are presented as n (% of non-missing). Missing are presented for variables with missing values as n (% of all) in italics.

b The subfertile groups are untreated subfertility, ovulation induction, and ART.

c Low birthweight (<2500 g) or preterm birth (<37 completed weeks gestation at delivery) recorded for this birth in mother’s primary care delivery data or HES maternity records.

**Figure 1.**
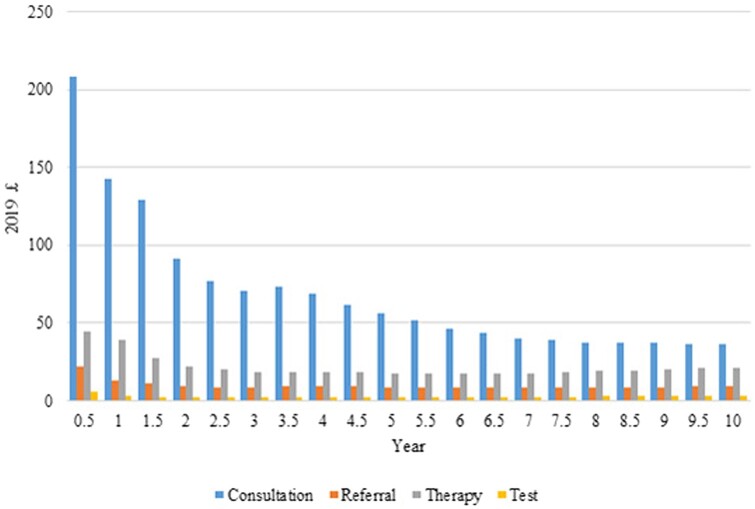
**Annual primary care costs (2019 £) per baby for the 10 years after birth, by type of service, for singletons in England**.

**Figure 2.**
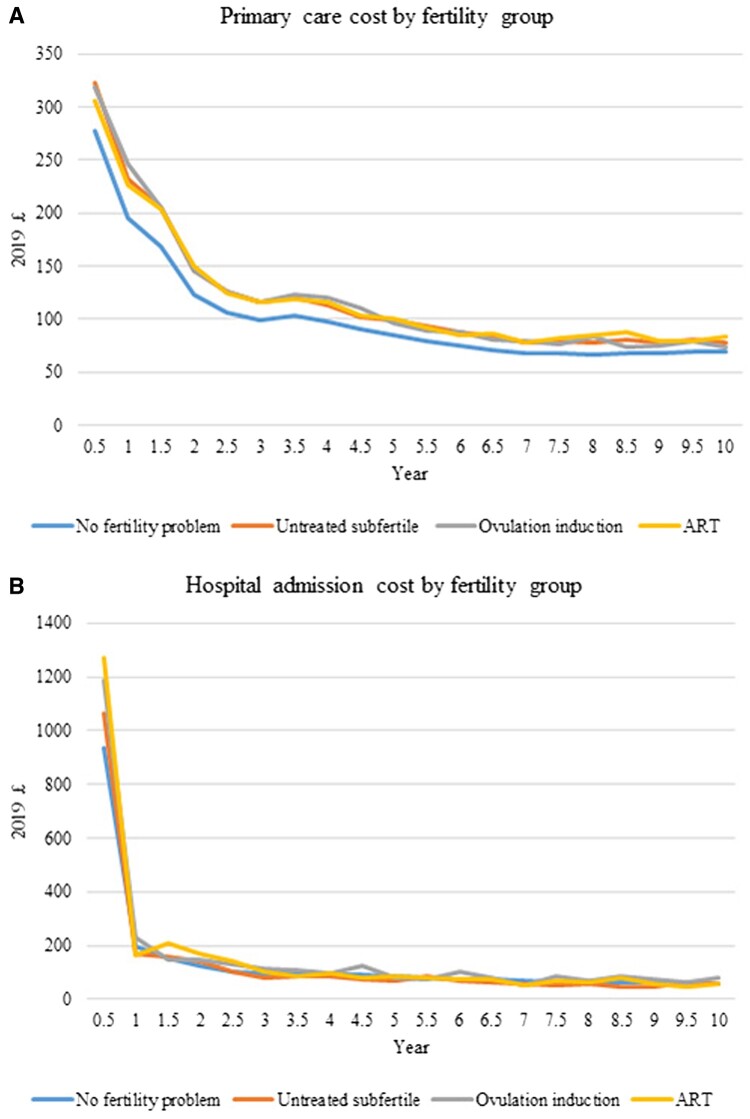
**Annual primary care and hospital admission costs (2019 £) per baby for the 10 years after birth, by fertility group for singletons in England**.

The observed trend in primary care and hospital costs per baby after birth is shown in Fig. 1 (primary care cost by type of services) and Fig. 2 (primary care and hospital costs by fertility group). Consultation costs was the major component for primary care costs (Fig. 1), with same pattern found in different fertility groups (Supplementary Fig. S4). Compared to children born to mothers with no fertility problems, children born after untreated sub-fertility, ovulation induction, or ART had higher primary care costs throughout the 10-years’ follow up (Fig. 2A), and higher hospital admission costs in the first six months after birth (Fig. 2B).

**Figure 3.**
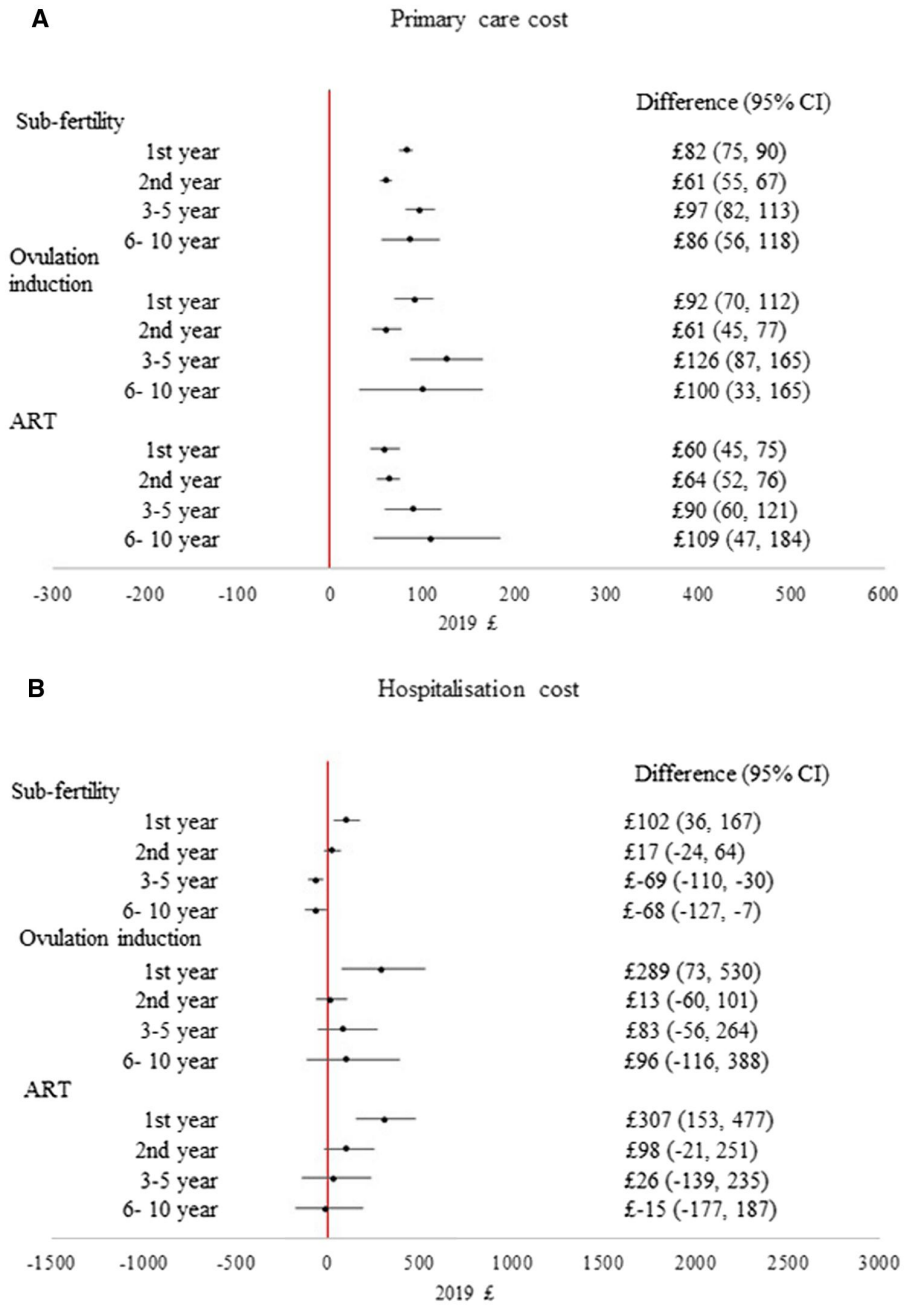
**Difference in adjusted primary care and hospital admission costs (2019 £) per baby between infertility groups and the comparison group, for singletons in England**.

The IPW-adjusted number of consultations and health costs in the 1st, 2nd, 3rd–5th, and 6th–10th years after birth in different groups are presented in Table 2, with the differences in costs between the group with no fertility problems (comparison group) and the sub-fertility or fertility treatment groups presented in Fig. 3A. Children born to mothers with no fertility problems had significantly fewer consultations and lower primary care costs compared to other groups throughout the 10-years’ follow up. Primary care costs and numbers of consultations in the three infertility groups were found to be similar. These results were similar when attrition was not accounted for (Supplementary Table S2).

**Table 2.** Average number of GP consultations per child, primary care cost, and hospital admission cost (2019 £) in the 1st, 2nd, 3rd?5th, and 6th?10th years after birth, by fertility group.

	No fertility problem		Untreated sub-fertility		Ovulation induction		ART	
	Mean	95% CI	Mean	95% CI	Mean	95% CI	Mean	95% CI
No. of consultations								
1st year	9.5	(9.5, 10.9)	10.9	(10.8, 10.9)	10.8	(10.5, 11)	10.5	(10.3, 10.7)
2nd year	5.9	(5.9, 6.9)	6.9	(6.8, 7)	6.9	(6.7, 7.2)	7.0	(6.8, 7.2)
3rd–5th years	10.4	(10.5, 11.9)	11.9	(11.8, 12.1)	12.8	(12.3, 13.3)	12.2	(11.8, 12.6)
6th–10th years	9.4	(9.4, 10.8)	10.8	(10.5, 11)	11.1	(10.3, 11.8)	11.0	(10.3, 11.6)
Primary care costs								
1st year	473	(475, 555)	555	(548, 563)	565	(543, 585)	533	(518, 549)
2nd year	291	(293, 352)	352	(347, 358)	353	(337, 368)	355	(343, 368)
3rd–5th years	573	(577, 670)	670	(655, 686)	699	(660, 738)	663	(634, 693)
6th–10th years	661	(669, 748)	748	(719, 777)	761	(697, 825)	771	(710, 844)
Hospital admission costs								
1st year	1129	(1111, 1148)	1231	(1168, 1293)	1418	(1200, 1661)	1436	(1283, 1606)
2nd year	282	(274, 290)	298	(258, 346)	295	(221, 384)	380	(262, 533)
3rd–5th years	570	(555, 587)	501	(464, 537)	653	(514, 834)	597	(437, 815)
6th–10th years	677	(659, 697)	609	(554, 667)	774	(565, 1063)	662	(506, 863)

IPW was used to adjust for attrition in CPRD data. GP, general practitioner.

Regarding hospital costs, children born after ART had a significantly higher hospital admission costs in the first year after birth compared to those born to women with no fertility problems (difference = £307 (95% CI: 153, 477)). The first-year hospital admission costs were also significantly higher among babies born after ovulation induction compared to the no fertility problem group (difference = £289 (95% CI: 73, 530)) and, to a lesser extent, among those born after untreated subfertility (difference = £102 (95% CI: 36, 167)) (Fig. 3B).

The results of the additional analyses were broadly consistent with the findings presented here. Findings for twins/triplets are provided in Supplementary Tables S3 and S4, and Supplementary Figs S5 and S6; the results show similar patterns although first year hospital costs are higher across all multiple births compared to singleton births. Results for primary care utilization and costs in all singleton births 1992–2017 without restricting to those with HES linkage, are provided in Supplementary Tables S5 and S6, and Supplementary Figs S7 and S8. As in the main analysis, primary care costs are higher in all infertility groups and do not differ substantially in those who did and did not receive treatment.

## Discussion

Using a large bespoke linked administrative dataset in England, this study investigated the longer-term healthcare utilization and costs for children born following maternal subfertility and fertility treatment. Compared to children born to mothers with no fertility problems, the children born after sub-fertility or born after ovulation induction or ART had significantly more GP consultations and higher primary care costs up to 10 years after birth. However, the mean difference per child was less than £100 across each time period which may not be perceived as substantial in absolute terms. No difference was seen in the pattern of primary care utilization across the untreated subfertility and fertility treatment groups. Significantly higher hospital admission costs were also found in the subfertility and fertility treatment groups compared to the comparison group in the first year of life However, after age 12 months, there is no evidence of an effect on all-cause hospitalization.

To our knowledge, this is the first detailed evaluation of the longer-term healthcare utilization and costs after ART in the UK healthcare system. A previous study assessed the long-term broader economic consequences of children born following ART from the perspective of the government and found that an investment of £12 931 to achieve an IVF singleton was worth 8.5 times this amount to the UK Treasury in discounted future tax revenue (Connolly *et al.*, 2009). A Finnish study compared post-neonatal hospitalization and costs up to 7 years of age between IVF children and control children and found that the incidence of multiple births increases the utilization of post-neonatal healthcare services and costs among IVF children, with increased hospitalization and costs also seen among IVF singletons (Koivurova *et al.*, 2007). This is slightly different from our findings, as we did not find evidence of higher hospital admission cost in the ART group after 12 months of age. The Finnish study was based on a cohort of IVF children born from 1990 to 1995, who might have had a different pattern of hospital admissions after birth compared to babies in our study (born 1997–2009 for the hospital sample), due to the ART techniques developing rapidly and a different source population. A recent US study evaluated differences in child healthcare utilization among singleton births by maternal fertility status in the first four years after birth (Dukhovny *et al.*, 2021). Dukhovny *et al.* found that those born following unassisted sub-fertility, medically assisted reproduction, and ART were more likely to have hospital-based care in their first 4 years. In our study the difference was only observed in the first year, and it is likely that much of the association could be attributed to differences in other characteristics such as prematurity. It is worth noting that this US study only included babies who had private medical insurance, while ours is based on the use of the National Health Service which is free at the point of access; other differences between the US and UK healthcare system also make it harder to directly compare the results of these two studies.

Our study also represents the first study incorporating primary care utilization into the evaluation of healthcare cost associated with ART in the longer term. We found that children born to mothers who experienced untreated subfertility or fertility treatment had more GP visits and higher primary care costs up to 10 years after birth compared to children born to mothers with no evidence of fertility problems, while for hospital admission costs there were no significant differences across the fertile and sub-fertile groups after the first year. The differences in all-cause hospitalization costs were strongly influenced by duration of the birth admission, and preterm or low birth weight babies. While the differences in hospitalization costs between the groups are reduced as the children grow up, there continues to be a higher cost associated with primary care consultations and treatment for children born after fertility problems. This increased primary care utilization and cost does not differ significantly across the groups of children born after different fertility problems, which suggests that any effect on the child’s health may be a result of the underlying parent’s fertility issues rather than the treatments itself.

Other research has indicated that there are significant health implications and associated increased costs of a multiple birth after IVF, compared to a singleton IVF birth (Ledger *et al.*, 2006; van Heesch *et al.*, 2015). As a result, single-embryo transfer has been suggested as an approach that may result in improved health outcomes and substantial savings to the healthcare system. In this study, we found that even singleton children born following ART still had significantly higher hospital admission costs in the first year after birth. Further investigation of the causes of these early life hospital admissions will help to elucidate what underpins these differences.

Our study has important implications for both the families and health service providers. Reassuringly, this study shows that children born following ART are not at an increased risk of hospitalization after the first year, which is consistent with the research evidence that indicates most children born after ART are healthy. This may help to advocate for the use of single embryo transfer among women with infertility problems, which is rapidly becoming the norm. On the other hand, as the demand for and use of fertility treatment continues to grow in the UK, it is important to consider the longer-term impact on health service provision and associated costs.

The strengths of this study include the use of a large sample size with long-term follow-up of both primary care and hospital admission utilization and costs estimated based on detailed staff, consultation, and hospital admission types. Linkage to HFEA register records of fertility treatment provided details of women’s fertility treatment history, and allowed more accurate estimation of exposure than did using primary care records alone. We used the IPW method to ensure that loss to follow up does not bias the findings. The comparison of treated and untreated fertility problems helps to elucidate whether any adverse effects are due to underlying fertility problems or the treatment of these problems.

The main limitation of the study is that it was based on routine records, which reflect not only the quality of the record keeping but also the healthcare-seeking behaviours of the patients. The untreated subfertility and ovulation induction groups were identified through routine primary care records, and so women needed to have consulted their GP about this issue to be identified. It may be that women who seek support for fertility issues are also more likely to utilize services for other health conditions for their children. However, this does not explain the increase in the costs associated with hospitalization, which tends to be less influenced by the parent’s choice to seek care. HFEA linkage is permitted for non-donor cycles only and after the introduction of consent for data use in 2009, only data for couples who gave consent can be linked. Recording of few personal identifiers in the HFEA data also means that linkage algorithms may not identify all matches. Therefore, the use of both primary care and HFEA records of ART to allocate conception history in this study improves the reliability of the exposure. CPRD mother–baby linked data require children to be registered with the GP, as consequence, stillbirths and neonatal deaths are likely to be missed. We have explored all-cause hospitalization and consultation data, which gives an overview of the impact at the population level of service utilization and costs, but which may mask cause-specific differences. It would be useful for future studies to explore the differences in the diagnosis and treatment of specific conditions within this population, and to investigate their role in the additional healthcare costs associated with conception after any fertility problems. We were not able to estimate cost of neonatal critical care in this study as such information is not captured in the HES APC data. This may cause underestimation on the costs between the comparison group and the infertility groups. Current practice puts an emphasis on single embryo transfer and we present findings for singletons only, however, the overall cost associated with fertility treatment including multiple births will be higher than these estimates.

In conclusion, in this study we quantified the 10-year healthcare utilization and costs for children associated with their parent’s subfertility and fertility treatments under the UK healthcare system. We found that compared to children born to mothers with no fertility problems, children born after fertility problems or fertility treatment had significantly more GP consultations and a small increase primary care costs up to 10 years after birth, as well as significantly higher hospital admission costs in the first year after birth. Parental subfertility, or factors associated with it, appears to underpin this association rather than an adverse effect of treatment. These findings can help anticipate the financial impact on the healthcare system associated with the care of children born after fertility problems including ART, and can inform the planning and provision of primary care and paediatric services.

## Supplementary Material

dead198_Supplementary_Figure_S1

dead198_Supplementary_Figure_S2

dead198_Supplementary_Figure_S3

dead198_Supplementary_Figure_S4

dead198_Supplementary_Figure_S5

dead198_Supplementary_Figure_S6

dead198_Supplementary_Figure_S7

dead198_Supplementary_Figure_S8

dead198_Supplementary_Table_S1

dead198_Supplementary_Table_S2

dead198_Supplementary_Table_S3

dead198_Supplementary_Table_S4

dead198_Supplementary_Table_S5

dead198_Supplementary_Table_S6

dead198_Supplementary_Materials_Methods

## Data Availability

The data underlying this article were accessed from the Clinical Practice Research Datalink with bespoke linkage to HFEA register data, and were used under licence with study-specific permission. The authors cannot share these data, but application can be made directly to CPRD and HFEA.
