# Supplementary material for: Long-term healthcare utilization and costs of babies born after assisted reproductive technologies (ART): a record linkage study with 10-years’ follow-up in England
Source: Hum Reprod. 2023 Oct 7;38(12):2507–15. doi: 10.1093/humrep/dead198 (PMC10694410; doi:10.1093/humrep/dead198)
Supplement: dead198_Supplementary_Figure_S1 [file dead198_supplementary_figure_s1.pdf]

Human Reproduction, 2023, 1–1
https://doi.org/10.1093/humrep/dead198
Supplementary Data

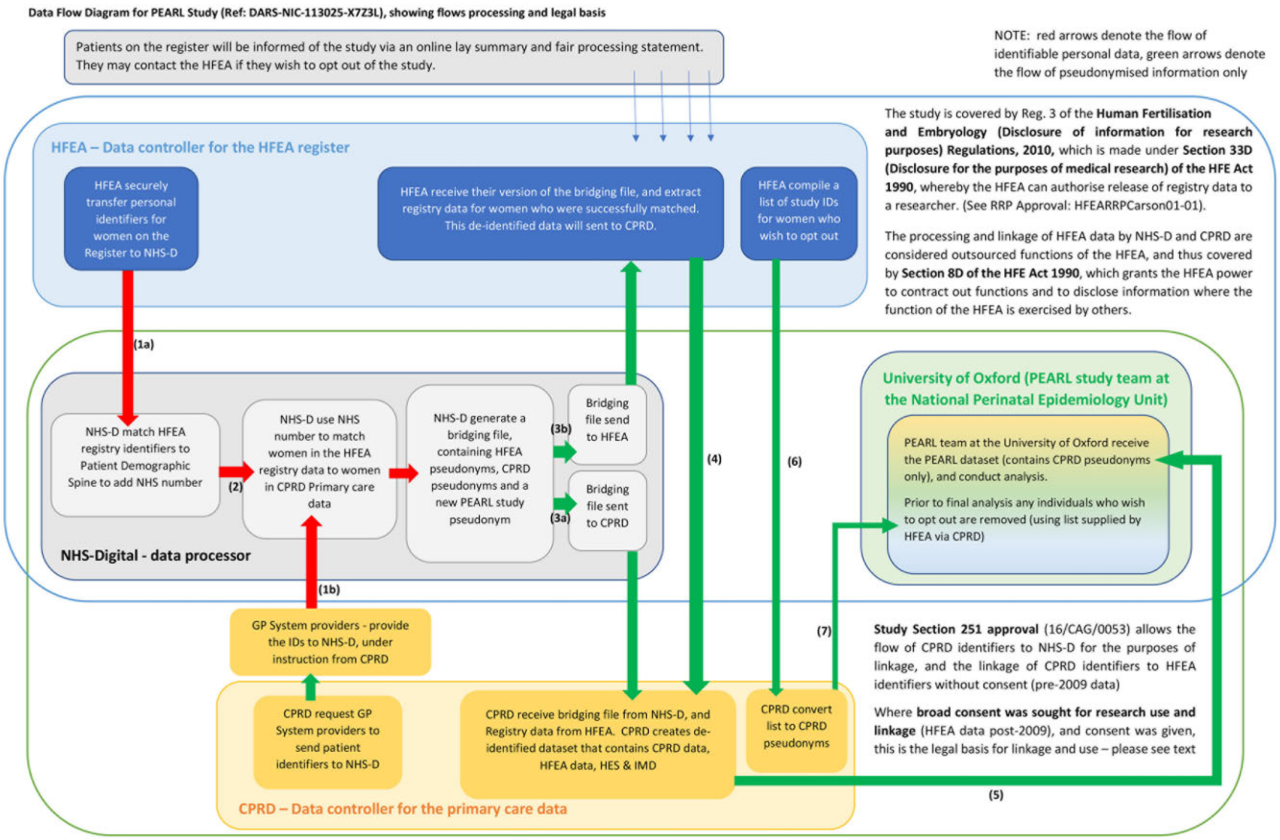

Supplementary Figure S1. Data flow diagram for the PEARL study linkage process, with legal basis for each data flow. CPRD, Clinical Practice Research Datalink; GP, general practitioner; HFEA, Human Fertilisation and Embryology Authority; ID, identifier; IMD, Indices of Multiple Deprivation; HES, Hospital Episode Statistics; NHS-D, NHS Digital (now NHS-England).
