# Supplementary material for: Long-term healthcare utilization and costs of babies born after assisted reproductive technologies (ART): a record linkage study with 10-years’ follow-up in England
Source: Hum Reprod. 2023 Oct 7;38(12):2507–15. doi: 10.1093/humrep/dead198 (PMC10694410; doi:10.1093/humrep/dead198)
Supplement: dead198_Supplementary_Figure_S3 [file dead198_supplementary_figure_s3.pdf]

OXFORD
human reproduction

*Human Reproduction*, 2023, 1–1
https://doi.org/10.1093/humrep/dead198
Supplementary Data

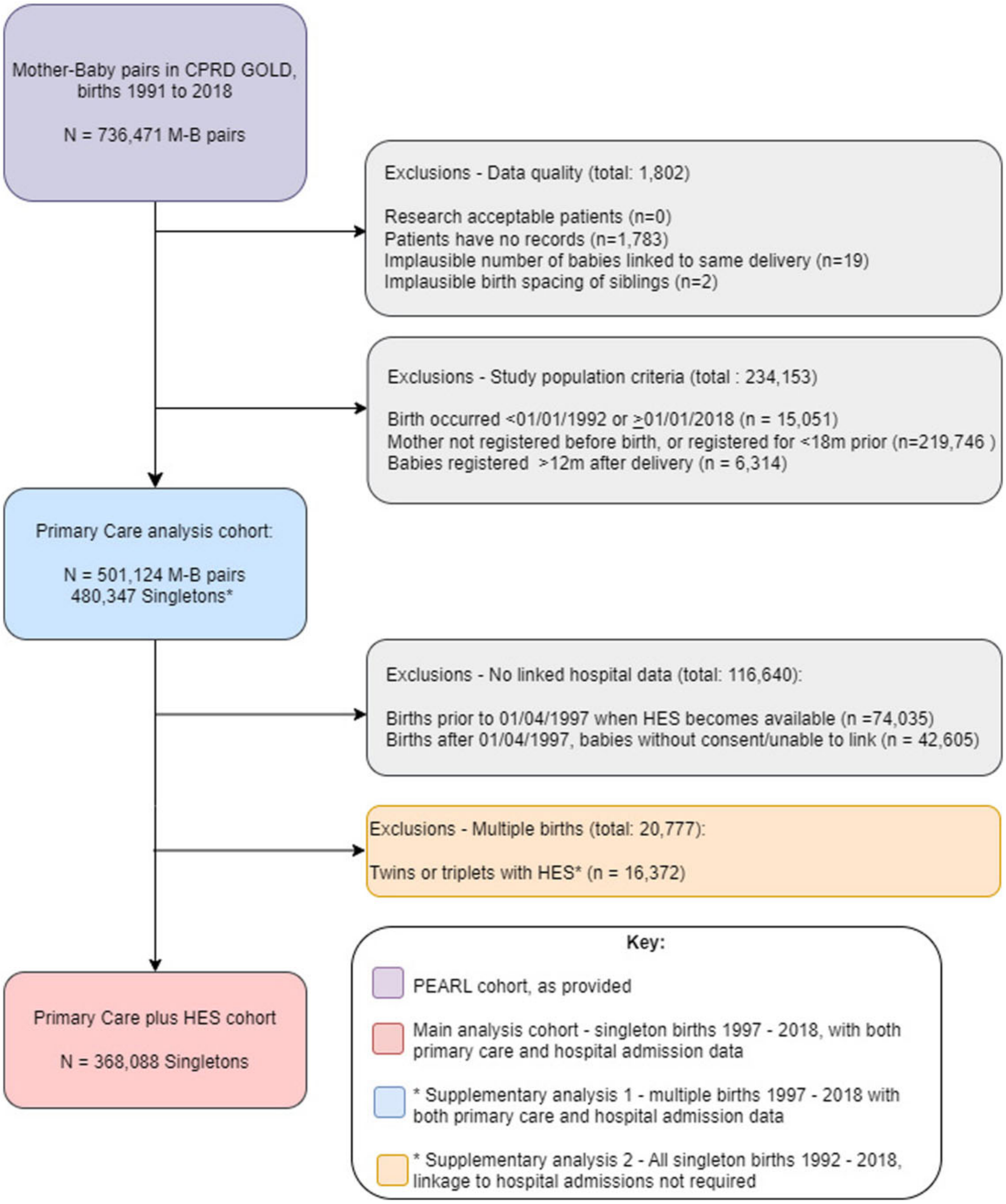


**Supplementary Figure S3. Flow chart for the study population.** CPRD, Clinical Practice Research Datalink; HES, Hospital Episode Statistics; M–B, mother–baby linked.
