# Supplementary material for: Long-term healthcare utilization and costs of babies born after assisted reproductive technologies (ART): a record linkage study with 10-years’ follow-up in England
Source: Hum Reprod. 2023 Oct 7;38(12):2507–15. doi: 10.1093/humrep/dead198 (PMC10694410; doi:10.1093/humrep/dead198)
Supplement: dead198_Supplementary_Figure_S8 [file dead198_supplementary_figure_s8.pdf]

Human Reproduction, 2023, 1–1
https://doi.org/10.1093/humrep/dead198
Supplementary Data

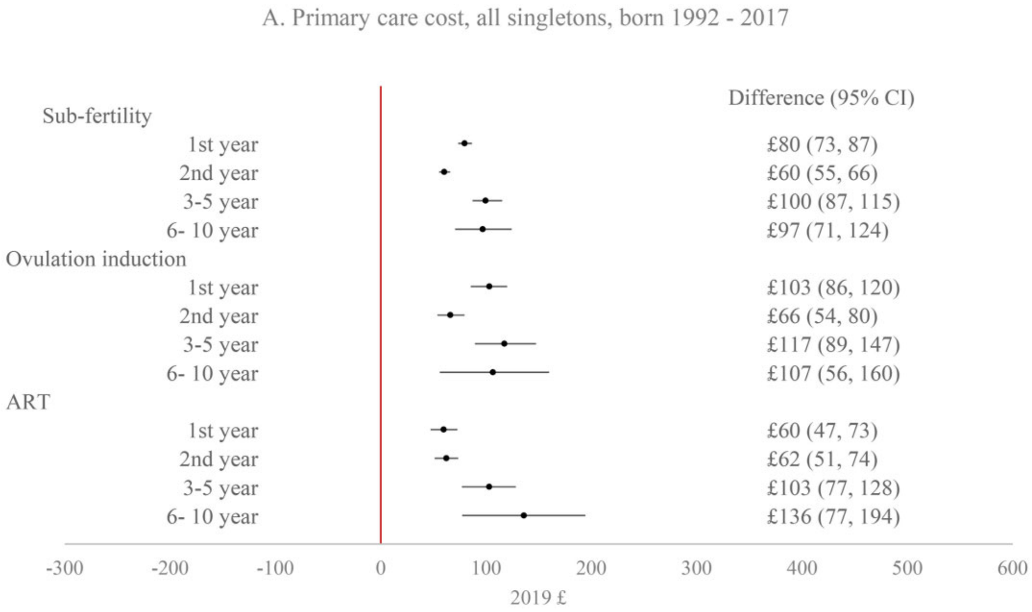

Supplementary Figure S8. Forest plot to show adjusted differences in cost compared to 'no fertility problem' group, for singletons born 1992–2017, no requirement for HES linkage. CI, confidence interval; HES, Hospital Episode Statistics.
