# Supplementary material for: Long-term healthcare utilization and costs of babies born after assisted reproductive technologies (ART): a record linkage study with 10-years’ follow-up in England
Source: Hum Reprod. 2023 Oct 7;38(12):2507–15. doi: 10.1093/humrep/dead198 (PMC10694410; doi:10.1093/humrep/dead198)
Supplement: dead198_Supplementary_Table_S1 [file dead198_supplementary_table_s1.pdf]

OXFORD
human reproduction

Human Reproduction, 2023, 1–2
https://doi.org/10.1093/humrep/dead198
Supplementary Data

Supplementary Table S1. Follow-up of (i) children in main sample, and additional analysis cohorts (ii) multiple births, born 1997–2017, with HES linkage, and (iii) singletons, born 1997–2017, no HES linkage required.

		Main analysis (singletons) N = 368 088 (100%)	Additional 1 (multiples) N = 16 372 (100%)	Additional 2 (Singletons, no HES) N = 480 347
Year 0	No. at risk	N=368 088	16 372 (100.0%)	480 342 (100.0%)
Year 0.5	No. at risk	352 324 (95.7%)	15 784 (96.4%)	461 977 (96.2%)
	No. of deaths	196 (0.1%)	13 (0.1%)	296 (0.1%)
	No. censored due to relocation	9716 (2.6%)	388 (2.4%)	11 347 (2.4%)
	No. censored due to study close	5852 (1.6%)	187 (1.1%)	6727 (1.4%)
Year 1	No. at risk	331 125 (90.0%)	14 976 (91.5%)	438 124 (91.2%)
	No. of deaths	275 (0.1%)	21 (0.1%)	410 (0.1%)
	No. censored due to relocation	22 211 (6.0%)	900 (5.5%)	26 025 (5.4%)
	No. censored due to study close	14 477 (3.9%)	475 (2.9%)	15 788 (3.3%)
Year 1.5	No. at risk	310 834 (84.4%)	14 144 (86.4%)	415 239 (86.4%)
	No. of deaths	332 (0.1%)	22 (0.1%)	480 (0.1%)
	No. censored due to relocation	33 555 (9.1%)	1420 (8.7%)	39 691 (8.3%)
	No. censored due to study close	23 367 (6.3%)	786 (4.8%)	24 937 (5.2%)
Year 2	No. at risk	291 750 (79.3%)	13 364 (81.6%)	393 572 (81.9%)
	No. of deaths	381 (0.1%)	24 (0.1%)	544 (0.1%)
	No. censored due to relocation	43 594 (11.8%)	1878 (11.5%)	52 015 (10.8%)
	No. censored due to study close	32 363 (8.8%)	1106 (6.8%)	34 216 (7.1%)
Year 2.5	No. at risk	273 660 (74.3%)	12 681 (77.5%)	372 983 (77.6%)
	No. of deaths	408 (0.1%)	24 (0.1%)	581 (0.1%)
	No. censored due to relocation	52 460 (14.3%)	2250 (13.7%)	63 126 (13.1%)
	No. censored due to study close	41 560 (11.3%)	1417 (8.7%)	43 657 (9.1%)
Year 3	No. at risk	257 137 (69.9%)	12 093 (73.9%)	354 109 (73.7%)
	No. of deaths	416 (0.1%)	24 (0.1%)	596 (0.1%)
	No. censored due to relocation	59 774 (16.2%)	2528 (15.4%)	72 557 (15.1%)
	No. censored due to study close	50 761 (13.8%)	1727 (10.5%)	53 085 (11.1%)
Year 3.5	No. at risk	240 727 (65.4%)	11 409 (69.7%)	335 359 (69.8%)
	No. of deaths	436 (0.1%)	24 (0.1%)	621 (0.1%)
	No. censored due to relocation	66 290 (18.0%)	2834 (17.3%)	81 108 (16.9%)
	No. censored due to study close	60 635 (16.5%)	2105 (12.9%)	63 259 (13.2%)
Year 4	No. at risk	225 443 (61.2%)	10 778 (65.8%)	317 855 (66.2%)
	No. of deaths	448 (0.1%)	24 (0.1%)	636 (0.1%)
	No. censored due to relocation	72 017 (19.6%)	3109 (19.0%)	88 743 (18.5%)
	No. censored due to study close	70 180 (19.1%)	2461 (15.0%)	73 113 (15.2%)
Year 4.5	No. at risk	211 186 (57.4%)	10 202 (62.3%)	301 533 (62.8%)
	No. of deaths	460 (0.1%)	25 (0.2%)	649 (0.1%)
	No. censored due to relocation	76 729 (20.8%)	3338 (20.4%)	95 237 (19.8%)
	No. censored due to study close	79 713 (21.7%)	2807 (17.1%)	82 928 (17.3%)
Year 5	No. at risk	197 999 (53.8%)	9723 (59.4%)	286 284 (59.6%)
	No. of deaths	470 (0.1%)	26 (0.2%)	661 (0.1%)
	No. censored due to relocation	80 753 (21.9%)	3506 (21.4%)	101 064 (21.0%)
	No. censored due to study close	88 866 (24.1%)	3117 (19.0%)	92 338 (19.2%)
Year 5.5	No. at risk	185 253 (50.3%)	9225 (56.3%)	271 639 (56.6%)
	No. of deaths	481 (0.1%)	27 (0.2%)	680 (0.1%)
	No. censored due to relocation	84 354 (22.9%)	3673 (22.4%)	106 310 (22.1%)
	No. censored due to study close	98 000 (26.6%)	3447 (21.1%)	101 718 (21.2%)
Year 6	No. at risk	173 726 (47.2%)	8783 (53.6%)	258 334 (53.8%)
	No. of deaths	490 (0.1%)	27 (0.2%)	692 (0.1%)
	No. censored due to relocation	87 359 (23.7%)	3804 (23.2%)	110 762 (23.1%)
	No. censored due to study close	106 513 (28.9%)	3758 (23.0%)	110 559 (23.0%)
Year 6.5	No. at risk	162 548 (44.2%)	8387 (51.2%)	245 484 (51.1%)
	No. of deaths	498 (0.1%)	28 (0.2%)	705 (0.1%)
	No. censored due to relocation	90 029 (24.5%)	3911 (23.9%)	114 775 (23.9%)
	No. censored due to study close	115 013 (31.2%)	4046 (24.7%)	119 383 (24.9%)
Year 7	No. at risk	151 938 (41.3%)	7979 (48.7%)	233 359 (48.6%)
	No. of deaths	500 (0.1%)	28 (0.2%)	710 (0.1%)
	No. censored due to relocation	92 416 (25.1%)	4018 (24.5%)	118 346 (24.6%)
	No. censored due to study close	123 234 (33.5%)	4347 (26.6%)	127 932 (26.6%)
Year 7.5	No. at risk	141 649 (38.5%)	7600 (46.4%)	221 631 (46.1%)
	No. of deaths	508 (0.1%)	28 (0.2%)	718 (0.1%)
	No. censored due to relocation	94 553 (25.7%)	4120 (25.2%)	121 592 (25.3%)
	No. censored due to study close	131 378 (35.7%)	4624 (28.2%)	136 406 (28.4%)

(continued)

Supplementary Table S1. (continued)

		Main analysis (singletons) N = 368 088 (100%)	Additional 1 (multiples) N = 16 372 (100%)	Additional 2 (Singletons, no HES) N = 480 347
Year 8	No. at risk	131 961 (35.9%)	7220 (44.1%)	210 456 (43.8%)
	No. of deaths	515 (0.1%)	28 (0.2%)	725 (0.2%)
	No. censored due to relocation	96 454 (26.2%)	4226 (25.8%)	124 578 (25.9%)
	No. censored due to study close	139 158 (37.8%)	4898 (29.9%)	144 588 (30.1%)
Year 8.5	No. at risk	122 783 (33.4%)	6886 (42.1%)	199 876 (41.6%)
	No. of deaths	525 (0.1%)	28 (0.2%)	738 (0.2%)
	No. censored due to relocation	98 182 (26.7%)	4331 (26.5%)	127 249 (26.5%)
	No. censored due to study close	146 598 (39.8%)	5127 (31.3%)	152 484 (31.7%)
Year 9	No. at risk	113 900 (30.9%)	6515 (39.8%)	189 644 (39.5%)
	No. of deaths	532 (0.1%)	28 (0.2%)	746 (0.2%)
	No. censored due to relocation	99 657 (27.1%)	4404 (26.9%)	129 634 (27.0%)
	No. censored due to study close	153 999 (41.8%)	5425 (33.1%)	160 323 (33.4%)
Year 9.5	No. at risk	105 341 (28.6%)	6229 (38.0%)	179 650 (37.4%)
	No. of deaths	536 (0.1%)	28 (0.2%)	753 (0.2%)
	No. censored due to relocation	101 036 (27.4%)	4467 (27.3%)	131 939 (27.5%)
	No. censored due to study close	161 175 (43.8%)	5648 (34.5%)	168 005 (35.0%)
Year 10	No. at risk	97 168 (26.4%)	5876 (35.9%)	170 161 (35.4%)
	No. of deaths	540 (0.1%)	29 (0.2%)	760 (0.2%)
	No. censored due to relocation	102 178 (27.8%)	4542 (27.7%)	133 930 (27.9%)
	No. censored due to study close	168 202 (45.7%)	5925 (36.2%)	175 496 (36.5%)

HES, Hospital Episode Statistics; No., number.
Analytical end date 20 February 2021.
