# Supplementary material for: Long-term healthcare utilization and costs of babies born after assisted reproductive technologies (ART): a record linkage study with 10-years’ follow-up in England
Source: Hum Reprod. 2023 Oct 7;38(12):2507–15. doi: 10.1093/humrep/dead198 (PMC10694410; doi:10.1093/humrep/dead198)
Supplement: dead198_Supplementary_Table_S2 [file dead198_supplementary_table_s2.pdf]

OXFORD
human reproduction
Human Reproduction, 2023, 1–1
https://doi.org/10.1093/humrep/dead198
Supplementary Data

Supplementary Table S2. Unadjusted number of consultations and primary care cost in 1st, 2nd, 3rd–5th, and 6th–10th years, by fertility group

	No fertility problems		Untreated subfertility		Ovulation induction		ART	
	**Mean**	**(95% CI)**	**Mean**	**(95% CI)**	**Mean**	**(95% CI)**	**Mean**	**(95% CI)**
Number of consultations								
1st year	9.5	(9.5, 9.5)	10.9	(10.8, 10.9)	10.8	(10.5, 11)	10.5	(10.3, 10.7)
2nd year	5.9	(5.9, 5.9)	6.9	(6.8, 7)	6.9	(6.7, 7.2)	7	(6.9, 7.2)
3rd–5th years	10.7	(10.6, 10.7)	12.2	(12, 11.8)	12.8	(12.4, 13.3)	12.5	(12.1, 12.8)
6th–10th years	10.2	(10.1, 10.2)	11.8	(11.6, 12)	11.8	(11.2, 12.4)	12	(11.5, 12.6)
Primary care costs								
1st year	473	(472, 475)	555	(548, 562)	564	(543, 584)	533	(518, 549)
2nd year	292	(290, 293)	351	(346, 357)	352	(336, 367)	354	(342, 367)
3rd–5th years	583	(580, 586)	678	(663, 693)	694	(658, 730)	681	(652, 712)
6th–10th years	705	(698, 711)	817	(788, 848)	798	(736, 866)	839	(780, 907)
