# Supplementary material for: Long-term healthcare utilization and costs of babies born after assisted reproductive technologies (ART): a record linkage study with 10-years’ follow-up in England
Source: Hum Reprod. 2023 Oct 7;38(12):2507–15. doi: 10.1093/humrep/dead198 (PMC10694410; doi:10.1093/humrep/dead198)
Supplement: dead198_Supplementary_Table_S3 [file dead198_supplementary_table_s3.pdf]

OXFORD
human reproduction
Human Reproduction, 2023, 1–1
https://doi.org/10.1093/humrep/dead198
Supplementary Data

Supplementary Table S3. Characteristics of multiples, born 1997–2017, with HES linkage.

	All multiples	No fertility problems	Untreated subfertility	Ovulation induction	ART	P-value (all groups)	P-value (SF, OI, ART only)
N (% of total)	16 372	13 481	1285	201	1405		
**Mum's characteristics**							
**Age at delivery**						<0.001	<0.001
<25	1886 (11.5%)	1829 (13.6%)	41 (3.2%)	9 (4.5%)	7 (0.5%)		
25–29	3463 (21.2%)	3059 (22.7%)	221 (17.2%)	53 (26.4%)	130 (9.3%)		
30–34	5552 (33.9%)	4455 (33.0%)	459 (35.7%)	79 (39.3%)	559 (39.8%)		
35–39	4269 (26.1%)	3269 (24.2%)	399 (31.1%)	50 (24.9%)	551 (39.2%)		
≥40	1202 (7.3%)	869 (6.4%)	165 (12.8%)	10 (5.0%)	158 (11.2%)		
Missing, n (%)	*0 (0.0)%*	*0.0%*	*0.0%*	*0.0%*	*0.0%*		
**Ethnicity**						0.46	0.28
White	3827 (46.5%)	3116 (46.5%)	323 (44.5%)	43 (51.2%)	345 (48.0%)		
Minority ethnic group	4398 (53.5%)	3580 (53.5%)	403 (55.5%)	41 (48.8%)	374 (52.0%)		
Missing, n (%)	*8147 (49.8%)*	*6785 (50.3%)*	*559 (43.5%)*	*117 (58.2%)*	*686 (48.8%)*		
**Smoking history**						<0.001	0.15
Current	2072 (30.6%)	1850 (33.0%)	118 (20.3%)	19 (26.0%)	85 (16.3%)		
Ex	1201 (17.7%)	957 (17.1%)	113 (19.4%)	12 (16.4%)	119 (22.9%)		
Never	3506 (51.7%)	2797 (49.9%)	351 (60.3%)	42 (57.5%)	316 (60.8%)		
Missing, n (%)	*9593 (58.6%)*	*7877 (58.4%)*	*703 (54.7%)*	*128 (63.7%)*	*885 (63%)*		
**BMI before pregnancy***							
Mean (SD)	25.9 (5.9)	26.1 (6.0)	24.8 (5.3)	27.0 (7.3)	24.8 (5.0)	<0.001	0.007
Missing, n (%)	*11 337 (69.2%)*	*9325 (69.2%)*	*852 (66.3%)*	*139 (69.2%)*	*1021 (72.7%)*		
**Child's characteristics**							
**Year of birth**							
1997–2003	6947 (42.4%)	6028 (44.7%)	355 (27.6%)	108 (53.7%)	456 (32.5%)	<0.001	<0.001
2004–2008	4048 (24.7%)	3234 (24.0%)	339 (26.4%)	49 (24.4%)	426 (30.3%)		
2009–2013	3885 (23.7%)	3035 (22.5%)	431 (33.5%)	34 (16.9%)	385 (27.4%)		
≥2014	1492 (9.1%)	1184 (8.8%)	160 (12.5%)	10 (5.0%)	138 (9.8%)		
Missing, n (%)	*0 (0%)*	*0 (0%)*	*0 (0%)*	*0 (0%)*	*0 (0%)*		
**Sex**							
Male	8028 (49.0%)	6657 (49.4%)	576 (44.8%)	108 (53.7%)	687 (48.9%)	0.009	0.019
Female	8344 (51.0%)	6824 (50.6%)	709 (55.2%)	93 (46.3%)	718 (51.1%)		
Missing, n (%)	*0 (0%)*	*0 (0%)*	*0 (0%)*	*0 (0%)*	*0 (0%)*		
**IMD (patient level)**							
Least deprived 1	4046 (24.7%)	3076 (22.8%)	418 (32.5%)	57 (28.4%)	495 (35.2%)	<0.001	0.16
2	3647 (22.3%)	2941 (21.8%)	307 (23.9%)	48 (23.9%)	351 (25.0%)		
3	3343 (20.4%)	2759 (20.5%)	258 (20.1%)	41 (20.4%)	285 (20.3%)		
4	3139 (19.2%)	2714 (20.2%)	201 (15.6%)	36 (17.9%)	188 (13.4%)		
Most deprived 5	2181 (13.3%)	1975 (14.7%)	101 (7.9%)	19 (9.5%)	86 (6.1%)		
Missing, n (%)	*16 (0.1%)*	*16 (0.1%)*	*0 (0%)*	*0 (0%)*	*0 (0%)*		
**IMD (practice level)**							
Least deprived 1	2537 (15.5%)	1986 (14.7%)	247 (19.2%)	34 (16.9%)	270 (19.2%)	<0.001	0.038
2	3510 (21.4%)	2766 (20.5%)	322 (25.1%)	42 (20.9%)	380 (27.0%)		
3	3934 (24.0%)	3306 (24.5%)	297 (23.1%)	56 (27.9%)	275 (19.6%)		
4	3190 (19.5%)	2654 (19.7%)	227 (17.7%)	31 (15.4%)	278 (19.8%)		
Most deprived 5	3201 (19.6%)	2769 (20.5%)	192 (14.9%)	38 (18.9%)	202 (14.4%)		
Missing, n (%)	*0 (0%)*	*0 (0%)*	*0 (0%)*	*0 (0%)*	*0 (0%)*		
**Low birthweight or preterm birth[$]**							
No	4499 (69.5%)	4016 (72.7%)	211 (49.1%)	50 (72.5%)	222 (49.2%)	<0.001	<0.001
Yes	1978 (30.5%)	1511 (27.3%)	219 (50.9%)	19 (27.5%)	229 (50.8%)		
Missing, n (%)	*9865 (60.3%)*	*7954 (59%)*	*855 (66.5%)*	*132 (65.7%)*	*954 (67.9%)*		

IMD, Index of Multiple Deprivation; OI, ovulation induction; SF, untreated subfertile.
[*] Continuous variables are presented as mean (standard deviation); category variables are presented as n (% of non-missing); missing are presented for variables with missing values as n (% of all) in *italics*.
[$] Low birthweight (<2500 g) or preterm birth (<37 completed weeks gestation at delivery) recorded for this birth in mother's primary care delivery data or HES maternity records.
