# Supplementary material for: Long-term healthcare utilization and costs of babies born after assisted reproductive technologies (ART): a record linkage study with 10-years’ follow-up in England
Source: Hum Reprod. 2023 Oct 7;38(12):2507–15. doi: 10.1093/humrep/dead198 (PMC10694410; doi:10.1093/humrep/dead198)
Supplement: dead198_Supplementary_Table_S4 [file dead198_supplementary_table_s4.pdf]

OXFORD human reproduction

Human Reproduction, 2023, 1–1
https://doi.org/10.1093/humrep/dead198
Supplementary Data

Supplementary Table S4. Primary care consultations, costs, and hospitalization costs in twins/triplets.

	No fertility problem	Untreated subfertility	Ovulation induction	ART
	**Mean (95% CI)**	**Mean (95% CI)**	**Mean (95% CI)**	**Mean (95% CI)**
**Primary care**				
Unadjusted				
No. of consultation				
1st year total	9.2 (9.1, 9.3)	9.9 (9.5, 10.2)	9.5 (8.7, 10.3)	9.4 (9.2, 9.7)
2nd year total	5.5 (5.4, 5.6)	6.4 (6.1, 6.7)	6 (5.4, 6.6)	6.2 (5.9, 6.5)
3rd–5th year total	10.1 (9.9, 10.2)	10.9 (10.8, 11)	11.8 (10.7, 13)	11 (10.4, 11.5)
6th–10th year total	9.7 (9.4, 9.9)	10.9 (10.1, 11.7)	9.8 (8, 11.7)	10.3 (9.6, 11.1)
Total primary care cost				
1st year total	524 (515, 533)	608 (579, 640)	570 (504, 635)	559 (533, 585)
2nd year total	281 (275, 287)	341 (316, 366)	299 (262, 334)	315 (295, 334)
3rd–5th year total	557 (543, 572)	618 (569, 673)	635 (558, 716)	598 (560, 640)
6th–10th year total	673 (646, 703)	706 (640, 776)	570 (464, 682)	727 (650, 823)
Adjusted (IPW)				
No. of consultation				
1st year total	9.2 (9.1, 9.3)	9.9 (9.5, 10.2)	9.5 (8.7, 10.3)	9.5 (9.2, 9.7)
2nd year total	5.5 (5.4, 5.6)	6.4 (6.1, 6.7)	6 (5.4, 6.7)	6.2 (5.9, 6.5)
3rd–5th year total	10 (9.8, 10.1)	10.8 (10.2, 11.4)	12 (10.7, 13.2)	10.9 (10.3, 12.6)
6th–10th year total	9.1 (8.8, 9.4)	10.7 (9.6, 11.9)	9.1 (7.7, 10.8)	11 (10.1, 12.5)
Total primary care cost				
1st year total	524 (516, 533)	609 (580, 641)	570 (504, 635)	559 (534, 587)
2nd year total	281 (275, 287)	342 (317, 369)	300 (262, 336)	316 (296, 336)
3rd–5th year total	555 (540, 571)	628 (569, 694)	649 (563, 736)	595 (558, 700)
6th–10th year total	647 (614, 685)	711 (631, 793)	569 (479, 673)	814 (695, 961)
**Hospital admissions**				
Unadjusted				
Total hospital cost				
1st year total	3156 (2991, 3317)	4433 (3888, 5097)	5183 (3471, 7458)	4224 (3662, 4821)
2nd year total	419 (365, 481)	369 (290, 448)	379 (198, 641)	455 (331, 595)
3rd–5th year total	682 (617, 748)	864 (536, 1414)	1275 (647, 2002)	786 (601, 1004)
6th–10th year total	859 (765, 960)	597 (489, 727)	879 (543, 1273)	772 (594, 981)

Note: IPW was used to adjust for attrition in CPRD data, and IPW was not required for HES analysis so only unadjusted results are presented.
IPW, inverse probability weight; No., number.
