# Supplementary material for: Long-term healthcare utilization and costs of babies born after assisted reproductive technologies (ART): a record linkage study with 10-years’ follow-up in England
Source: Hum Reprod. 2023 Oct 7;38(12):2507–15. doi: 10.1093/humrep/dead198 (PMC10694410; doi:10.1093/humrep/dead198)
Supplement: dead198_Supplementary_Table_S5 [file dead198_supplementary_table_s5.pdf]

OXFORD
human reproduction

Human Reproduction, 2023, 1–1
https://doi.org/10.1093/humrep/dead198
Supplementary Data

Supplementary Table S5. Characteristics of singletons, born 1992–2017, no requirement for HES linkage.

	All singletons	No fertility problems	Untreated subfertility	Ovulation induction	ART	P-value (all groups)	P-value (SF, OI, ART only)
N (% of total)	480 347	448 003	23 303	3227	5814		
**Mum's characteristics**							
**Age at delivery**							
<25	84 905 (17.7%)	82 927 (18.5%)	1731 (7.4%)	180 (5.6%)	67 (1.2%)	<0.001	<0.001
25–29	123 718 (25.8%)	117 434 (26.2%)	4906 (21.1%)	792 (24.5%)	586 (10.1%)		
30–34	156 727 (32.6%)	145 168 (32.4%)	8352 (35.8%)	1303 (40.4%)	1904 (32.7%)		
35–39	92 690 (19.3%)	83 348 (18.6%)	6265 (26.9%)	738 (22.9%)	2339 (40.2%)		
≥40	22 307 (4.6%)	19 126 (4.3%)	2049 (8.8%)	214 (6.6%)	918 (15.8%)		
Missing, n (%)	*0 (0.0%)*	*0 (0.0%)*	*0 (0.0%)*	*0 (0.0%)*	*0 (0.0%)*		
**Ethnicity**							
White	116 120 (48.6%)	108 132 (48.8%)	5883 (45.8%)	660 (46.0%)	1445 (47.0%)	<0.001	0.48
Minority ethnic	122 756 (51.4%)	113 398 (51.2%)	6955 (54.2%)	776 (54.0%)	1627 (53.0%)		
Missing, n (%)	*241 471 (50.3%)*	*226 473 (50.6%)*	*10 465 (44.9%)*	*1791 (55.5%)*	*2742 (47.2%)*		
**Smoking history**							
Current	67 071 (32.5%)	63 669 (33.1%)	2764 (25.5%)	273 (23.6%)	365 (17.5%)	<0.001	<0.001
Ex	31 577 (15.3%)	28 923 (15.1%)	1996 (18.4%)	176 (15.2%)	482 (23.1%)		
Never	107 508 (52.1%)	99 499 (51.8%)	6065 (56.0%)	708 (61.2%)	1236 (59.3%)		
Missing, n (%)	*274 191 (57.1%)*	*255 912 (57.1%)*	*12 478 (53.5%)*	*2070 (64.1%)*	*3731 (64.2%)*		
**BMI before pregnancy***							
Mean (SD)	25.4 (5.7)	25.4 (5.7)	25.7 (5.8)	26.9 (6.5)	24.9 (4.7)	<0.001	<0.001
Missing, n (%)	*321 438 (66.9%)*	*300 468 (67.1%)*	*14 560 (62.5%)*	*2179 (67.5%)*	*4231 (72.8%)*		
**Child's characteristics**							
**Year of birth**							
<1997	68 105 (14.2%)	64 512 (14.4%)	2271 (9.7%)	643 (19.9%)	679 (11.7%)	<0.001	<0.001
1997–2003	131 941 (27.5%)	124 250 (27.7%)	5072 (21.8%)	1075 (33.3%)	1544 (26.6%)		
2004–2008	118 088 (24.6%)	109 873 (24.5%)	6126 (26.3%)	752 (23.3%)	1337 (23.0%)		
2009–2013	116 480 (24.2%)	107 480 (24.0%)	6889 (29.6%)	622 (19.3%)	1489 (25.6%)		
≥2014	45 733 (9.5%)	41 888 (9.3%)	2945 (12.6%)	135 (4.2%)	765 (13.2%)		
Missing, n (%)	*0 (0.0%)*	*0 (0.0%)*	*0 (0.0%)*	*0 (0.0%)*	*0 (0.0%)*		
**Sex**							
Male	246 407 (51.3%)	229 789 (51.3%)	12 045 (51.7%)	1623 (50.3%)	2950 (50.7%)	0.73	0.19
Female	233 938 (48.7%)	218 212 (48.7%)	11 258 (48.3%)	1604 (49.7%)	2864 (49.3%)		
Missing, n (%)	*2 (0.0%)*	*2 (0.0%)*	*0 (0.0%)*	*0 (0.0%)*	*0 (0.0%)*		
**IMD (patient level)**							
Least deprived 1	107 561 (22.6%)	98 277 (22.1%)	6401 (27.7%)	934 (29.2%)	1949 (33.9%)	<0.001	<0.001
2	101 191 (21.2%)	93 571 (21.1%)	5319 (23.0%)	739 (23.1%)	1562 (27.2%)		
3	91 504 (19.2%)	85 464 (19.2%)	4363 (18.9%)	653 (20.4%)	1024 (17.8%)		
4	97 101 (20.4%)	91 645 (20.6%)	4102 (17.8%)	546 (17.1%)	808 (14.0%)		
Most deprived 5	78 883 (16.6%)	75 231 (16.9%)	2918 (12.6%)	326 (10.2%)	408 (7.1%)		
Missing, n (%)	*4107 (0.9%)*	*3815 (0.9%)*	*200 (0.9%)*	*29 (0.9%)*	*63 (1.1%)*		
**IMD (practice level)**							
Least deprived 1	71 801 (15.1%)	65 833 (14.8%)	4230 (18.3%)	504 (15.7%)	1234 (21.4%)	<0.001	<0.001
2	99 965 (21.0%)	92 490 (20.8%)	5300 (22.9%)	671 (21.0%)	1504 (26.1%)		
3	94 600 (19.8%)	88 152 (19.8%)	4551 (19.7%)	788 (24.6%)	1109 (19.3%)		
4	104 445 (21.9%)	98 140 (22.1%)	4662 (20.2%)	590 (18.4%)	1053 (18.3%)		
Most deprived 5	105 835 (22.2%)	99 953 (22.5%)	4380 (18.9%)	648 (20.2%)	854 (14.8%)		
Missing, n (%)	*3701 (0.8%)*	*3435 (0.8%)*	*180 (0.8%)*	*26 (0.8%)*	*60 (1.0%)*		
**Low birthweight or preterm birth$**							
No	307 219 (93.1%)	285 662 (93.2%)	16 158 (92.4%)	1826 (91.6%)	3573 (90.5%)	<0.001	<0.001
Yes	22 725 (6.9%)	20 847 (6.8%)	1333 (7.6%)	168 (8.4%)	377 (9.5%)		
Missing, n (%)	*150 403 (31.3%)*	*141 494 (31.6%)*	*5812 (24.9%)*	*1233 (38.2%)*	*1864 (32.1%)*		

IMD, Index of Multiple Deprivation; OI, ovulation induction; SF, untreated subfertile.
* Continuous variables are presented as mean (standard deviation); category variables are presented as n (% of non-missing); missing are presented for variables with missing values as n (% of all) in *italics*.
$ Low birthweight (<2500 g) or preterm birth (<37 completed weeks gestation at delivery).
