# Supplementary material for: Long-term healthcare utilization and costs of babies born after assisted reproductive technologies (ART): a record linkage study with 10-years’ follow-up in England
Source: Hum Reprod. 2023 Oct 7;38(12):2507–15. doi: 10.1093/humrep/dead198 (PMC10694410; doi:10.1093/humrep/dead198)
Supplement: dead198_Supplementary_Table_S6 [file dead198_supplementary_table_s6.pdf]

OXFORD

human reproduction

Human Reproduction, 2023, 1–1
https://doi.org/10.1093/humrep/dead198
Supplementary Data

Supplementary Table S6. Primary care consultations and primary care costs—singletons, 1992–2017, no HES required.

	No fertility problem	Untreated subfertility	Ovulation induction	ART
	Mean (95% CI)	Mean (95% CI)	Mean (95% CI)	Mean (95% CI)
**Unadjusted**				
No. of consultation				
1st year total	9.4 (9.4, 9.4)	10.8 (10.7, 10.9)	10.8 (10.6, 11.1)	10.4 (10.3, 10.6)
2nd year total	5.7 (5.7, 5.8)	6.8 (6.7, 6.9)	6.8 (6.6, 7)	6.9 (6.7, 7)
3rd–5th year total	10.5 (10.5, 10.6)	12.1 (11.9, 11.9)	12.7 (12.3, 13)	12.4 (12.1, 12.7)
6th–10th year total	9.9 (9.8, 9.9)	11.6 (11.3, 11.8)	11.7 (11.2, 12.2)	11.9 (11.5, 12.4)
Total primary care cost				
1st year total	475 (474, 477)	555 (549, 562)	578 (560, 595)	534 (522, 548)
2nd year total	292 (291, 293)	351 (346, 357)	357 (345, 370)	353 (341, 364)
3rd–5th year total	581 (578, 584)	680 (667, 693)	692 (665, 720)	693 (667, 720)
6th–10th year total	658 (653, 663)	778 (755, 804)	775 (728, 830)	825 (773, 881)
**Adjusted (IPW)**				
No. of consultation				
1st year total	9.4 (9.4, 9.4)	10.8 (10.7, 10.9)	10.8 (10.6, 11.1)	10.4 (10.3, 10.6)
2nd year total	5.7 (5.7, 5.7)	6.8 (6.7, 6.9)	6.8 (6.7, 7)	6.9 (6.7, 7)
3rd–5th year total	10.4 (10.3, 10.4)	11.9 (11.8, 12.1)	12.6 (12.3, 13)	12.2 (11.9, 12.6)
6th–10th year total	9.4 (9.3, 9.4)	10.8 (10.6, 11.1)	11.1 (10.6, 11.6)	11.1 (10.6, 11.6)
Total primary care cost				
1st year total	475 (474, 476)	555 (549, 562)	578 (561, 595)	535 (522, 548)
2nd year total	292 (290, 293)	352 (347, 357)	358 (345, 371)	354 (342, 365)
3rd–5th year total	573 (570, 576)	673 (660, 689)	691 (663, 721)	676 (650, 701)
6th–10th year total	643 (638, 650)	740 (715, 767)	750 (700, 804)	779 (723, 836)

Note: IPW was used to adjust for attrition in CPRD data. Hospitalizations were not analyzed as data were not available for all babies.
No., number.
