# Supplementary material for: Long-term healthcare utilization and costs of babies born after assisted reproductive technologies (ART): a record linkage study with 10-years’ follow-up in England
Source: Hum Reprod. 2023 Oct 7;38(12):2507–15. doi: 10.1093/humrep/dead198 (PMC10694410; doi:10.1093/humrep/dead198)
Supplement: dead198_Supplementary_Materials_Methods [file dead198_supplementary_materials_methods.docx]

**Supplementary Materials and Methods**

1. **Summary of linkage process**
2. **The linkage process (Please read with accompanying Supplementary Figure 1)**

The PEARL study is designed to assess the impact of successful fertility treatment on the long-term health of women and their children in England. To do this, **a new bespoke linked dataset was required** which links information on fertility treatment held in the Human Fertility and Embryology Authority Register to health data held in the Clinical Practice Research Datalink (comprising primary care data, and linked to Hospital Episode Statistics and Index of Multiple Deprivation).

The diagram lays out the data flow and processing steps. HFEA securely transfered personal identifiers for the women on their register to NHS Digital (NHS-D) (the disclosable data included were full name, date of birth, postcode (where available), NHS number (where available), HFEA patient ID number). NHS-D used these variables to match to women on the Patient Demographic Spine (MRIS) to add NHS number where it is not available on the register.

At the same time, CPRD requested that the GP system providers securely transfer personal identifiers to NHS-D (the disclosable data included were NHS number date of birth, gender, postcode, practice ID, patient ID). Then NHS-D matched women in the two files (CPRD IDs and HFEA IDs) using NHS number to create a bridging file. This bridging file contained only HFEA pseudonyms (patient ID number), CPRD pseudonyms (pracid, patid) and a new PEARL study ID pseudonym. The bridging file was split, and HFEA was sent a file containing HFEA pseudonym and PEARL pseudonym, while CPRD were sent CPRD pseudonym and PEARL pseudonym. Note, neither organisation receive the others pseudonym IDs.

The HFEA then extracted the registry data for the women identified in their bridging file, added the PEARL ID, stripped out all personal (disclosable) data and securely transferred this processed file to CPRD.

Using the PEARL ID supplied in the bridging file CPRD linked the HFEA data to their CPRD data and create a new, bespoke linked dataset. PEARL ID was removed, and the bespoke dataset was securely transferred to the PEARL study team at the University of Oxford (NPEU).

During this process (and for at least 6 months in total), the study was advertised and information was provided about how individuals on the HFEA register could opt out if they wish.

1. **Legal basis for the PEARL linkage study**

The **legal basis** for the study comprises four components:

1. The **flow of data** from the HFEA to the University of Oxford for this study is covered by **Regulation 3 of the Human Fertilisation and Embryology (Disclosure of Information for Research Purposes) Regulations 2010,** which is made under **Section 33D (Disclosure for the purposes of medical research) of the HFE Act, 1990,** whereby the HFEA can authorise the release of registry data to a researcher. This authorisation is in place, as is shown by the Register Research Panel approval (ref: HFEARRPCarson01-01).
2. The **processing** of the HFEA data by NHS-D and CPRD are considered outsourced functions of the HFEA, and thus are covered by **Section 8D of the HFE Act (1990),** which grants the HFEA the power to contract out functions and disclose information where the function of the HFEA is exercised by others.
3. Study specific **Section 251 approval (ref: 16/CAG/0053)** allows the flow of CPRD identifiers to NHS-D for the purposes of linkage, and the linkage of CPRD identifiers to HFEA identifiers **without consent** (this applies to pre-2009 HFEA data, as consent was not sought prior to 2010).
4. Where broad **consent for research use** was sought (HFEA data since 2009), and consent was given, this is the legal basis for the linkage of HFEA data to CPRD and use in this study. The consent forms are provided with this application, and while we recognise that they do not fulfil the incoming GDPR requirements we believe that the consent still forms a sufficient legal basis. The governance landscape has changed since these consent forms were drafted, but we believe that there would be a reasonable expectation among those who signed the consent forms that people who work with the databases (which *are* mentioned) would use identifiers for the linkage process. It is not possible to contact all those who consented to the use of data to update their consents (nor would it be appropriate, since they consented to **non-contact** research use of their data). In addition, the disclosure of information about a patient without their express consent may be justifiable, if the public interest in disclosing the information outweighs the patient’s interests in keeping it confidential. The identifiable data that is used in processing and linkage are held only by the HFEA and by NHS-D who have the highest possible patient confidentiality procedures and therefore the risk to patients is minimal. Identifiable data will not be released to either the CPRD or the study team at the University of Oxford. The use of the anonymised data will provide robust scientific evidence regarding the long term health outcomes for women and children after fertility treatment, which is in the public interest (as evidenced by the provision of Section 251 for the data for which there is not consent available).

**Please refer to the diagram, as each flow and process is numbered, and the legal basis for each is detailed below.**

**For individual flows and processing:**

1. The **release and flow of personal identifiable information** from HFEA register to NHS-D is made under Regulation 3 of Human Fertilisation and Embryology (Disclosure of Information for Research purposes) Regulations (2010) (which were made under Section 33D, Human Fertilisation and Embryology Act, 2010, amended 2008) and valid following approval from the HFEA RRP.
2. The **processing of HFEA personal identifiable information** by NHS-D to add NHS number is considered an outsourced function of the HFEA. Legal basis is Section 8D (1990 HFE Act, amended 2008).
3. The **flow of personal identifiable information** (CPRD identifiers) from GP service providers to NHS-D is covered by PEARL Section 251
4. The **processing and linkage of the CPRD and HFEA identifiers** to create a bridging file is covered by PEARL S251 (for pre-2009 data) and consent (for post-2009 data)
5. The **flow of the pseudonymised data** in the bridging file from NHS-D to HFEA is covered by Section 8D (1990 HFE Act, amended 2008).
6. The **flow of the pseudonymised data** in the bridging file from NHS-D to CPRD is covered by PEARL Section 251
7. The **flow of pseudonymised registry data** from HFEA to CPRD is covered by Section 8D (1990 HFE Act, amended 2008).
8. The **linkage of the pseudonymised HFEA data** to the pseudonymised CPRD dataset is covered by Section 8D (1990 HFE Act, amended 2008).
9. The **flow of the pseudonymised linked dataset** (CPRD+HFEA) to University of Oxford is covered by PEARL S251
10. The **flow of pseudonyms for opt outs** from HFEA to CPRD is covered by Section 8D
11. The **processing of pseudonyms** by CPRD to convert HFEA pseudonyms to CPRD pseudonyms using the bridging file is covered by consent (when the individuals request opt out, they give consent to use their personal information to find pseudonym on the bridging file, and to send the pseudonym to CPRD).
12. The **flow of pseudonyms** from CPRD to university of Oxford to apply these opt outs is covered by consent (when the individuals request opt out, they give consent to use their personal information to find pseudonym on the bridging file, and to send the pseudonym to CPRD, and for CPRD to send to University of Oxford).

**Legal basis under General Data Protection Regulations**

The legal basis for the processing and storage of personal data for PEARL is that it is ‘a task in the public interest’ (article 6(e)) and that sensitive personal data is necessary for archiving purposes in the public interest, scientific or historical research purposes or statistical purposes (article 9 (2) (j), based on Article 89(1)). This is particularly pertinent to the use of personal identifiers for the linkage by NHS-Digital. The dataset released to the research team is ‘pseudonymised’, meaning that they have no access to personal/sensitive personal data such as name, date of birth or NHS number, however, the same basis under GDPR applies to the pseudonymised linked dataset.

1. **Codes used to identify fertility groups in primary care data**

Each mother’s fertility history constructed from her clinical (consultation), prescription, test and referral records.

| 2.1 Female fertility problems | | |  |  |
| --- | --- | --- | --- | --- |
| Medcode | **Read code** | **Description** |  |  |
| 30392 | K5Bz.00 | Female infertility NOS |  |  |
| 4977 | K5B0.00 | Female infertility of anovulatory origin |  |  |
| 63421 | K5B0z00 | Female infertility of anovulatory origin NOS |  |  |
| 104569 | K5B7.00 | Female infertility due to diminished ovarian reserve |  |  |
| 62698 | K5B4.00 | Female infertility of cervical origin |  |  |
| 62084 | K5B1z00 | Female infertility of pituitary - hypothalamic cause NOS |  |  |
| 69884 | K5B1.00 | Female infertility of pituitary - hypothalamic origin |  |  |
| 35074 | K5B2.00 | Female infertility of tubal origin |  |  |
| 25077 | K5B2z00 | Female infertility of tubal origin NOS |  |  |
| 61299 | K5B3.00 | Female infertility of uterine origin |  |  |
| 68664 | K5B3z00 | Female infertility of uterine origin NOS |  |  |
| 48461 | K5B5.00 | Female infertility of vaginal origin |  |  |
| 41692 | 3189100 | Female infertility test abnormal |  |  |
| 17756 | 3189000 | Female infertility test normal |  |  |
| 33458 | 8C82.00 | Female infertility therapy |  |  |
| 9133 | 7E29000 | Fertility investigation of female NEC |  |  |
| 30785 | 12F3.00 | FH: Female infertility |  |  |
| 32199 | 1597.11 | H/O: female infertility |  |  |
| 16376 | 1597 | H/O: infertility - female |  |  |
| 1808 | K5B..00 | Infertility - female |  |  |
| 36264 | K26y200 | Infertility due to efferent duct obstruction |  |  |
| 16131 | 3189.11 | Infertility investigation -fem |  |  |
| 1154 | 3189 | Infertility investigations NOS |  |  |
| 36458 | K5By.00 | Other female infertility |  |  |
| 53018 | K5Byz00 | Other female infertility NOS |  |  |
| 6352 | C16..00 | Ovarian dysfunction |  |  |
| 44854 | C16z.00 | Ovarian dysfunction NOS |  |  |
| 47232 | PJ62.00 | Ovarian dysgenesis |  |  |
| 23802 | C163.11 | Ovarian hypogonadism |  |  |
| 35705 | K40..00 | Ovarian, fallopian tube and pelvic inflammatory diseases |  |  |
| 94385 | S77v100 | Ovary injury without mention of open wound into cavity |  |  |
| 17608 | 33A..00 | Ovulation test - temp. chart |  |  |
| 52132 | K5B0000 | Primary anovulatory infertility |  |  |
| 97461 | K5B4000 | Primary cervical infertility |  |  |
| 31030 | C163000 | Primary ovarian failure |  |  |
| 60861 | K5B2000 | Primary tubal infertility |  |  |
| 91280 | K5B3000 | Primary uterine infertility |  |  |
| 50116 | K5B0100 | Secondary anovulatory infertility |  |  |
| 69324 | K5B4100 | Secondary cervical infertility |  |  |
| 45985 | K5B2100 | Secondary tubal infertility |  |  |
| 73151 | K5B3100 | Secondary uterine infertility |  |  |
| 96463 | K5B5100 | Secondary vaginal infertility |  |  |

| 2.2 Male factor infertility (may be in women's notes as reasons for fertility issues) | | | | |
| --- | --- | --- | --- | --- |
| Medcode | **Read code** | **Description** |  |  |
| 93189 | ZG91100 | Advice on male subfertility |  |  |
| 54282 | K5B6.00 | Female infertility associated with male factors |  |  |
| 23900 | 7C26000 | Fertility investigation of male NEC |  |  |
| 45005 | 1AZ5.00 | Fertility problems in partner |  |  |
| 38160 | 12F2.00 | FH: Male infertility |  |  |
| 18196 | 14E2.00 | H/O: infertility - male |  |  |
| 39817 | 14E2.11 | H/O: male infertility |  |  |
| 99607 | K26y.00 | Infertility due to extratesticular cause |  |  |
| 58858 | K26yz00 | Infertility due to extratesticular cause NOS |  |  |
| 11189 | 3195.11 | Infertility investigation-male |  |  |
| 766 | K26..00 | Male infertility |  |  |
| 5292 | K26z.00 | Male infertility NOS |  |  |
| 21310 | 3195 | Male infertility testing |  |  |
| 43797 | 8C83.00 | Male infertility therapy |  |  |
| 29510 | 4912 | Semen exam.: good fertility |  |  |
| 7273 | 4916 | Semen exam.: infertile |  |  |
| 38990 | 4914 | Semen exam.: low fertility |  |  |
| 43258 | 4913 | Semen exam.:moderate fertility |  |  |
| 61954 | 4915 | Semen exam.:very low fertility |  |  |
|  |  |  |  |  |

| 2.3 General infertility codes and indicators of subfertility | | | | |
| --- | --- | --- | --- | --- |
| Medcode | **Read code** | **Description** |  |  |
| 1943 | K5By100 | Secondary infertility unspecified |  |  |
| 2014 | K5By000 | Primary infertility unspecified |  |  |
| 2957 | 1AZ2.11 | Infertility problem |  |  |
| 4571 | 9N07.00 | Seen in fertility clinic |  |  |
| 2949 | 9N1y600 | Seen in fertility clinic |  |  |
| 5239 | 8HTB.00 | Referral to fertility clinic |  |  |
| 7246 | K5Byz11 | Subfertility |  |  |
| 7351 | 1AZ2.00 | Fertility problem |  |  |
| 9036 | 6778.11 | Fertility counselling |  |  |
| 9938 | ZV26.00 | [V]Infertility management |  |  |
| 10205 | ZG9..00 | Advice relating to pregnancy and fertility |  |  |
| 10445 | ZG91.00 | Advice on fertility and infertility |  |  |
| 25307 | Z4P..00 | Reproductive counselling |  |  |
| 25361 | Z4P1.00 | Fertility counselling |  |  |
| 26088 | ZV26200 | [V]Infertility investigation and testing |  |  |
| 26150 | ZV26y00 | [V]Other specified infertility management |  |  |
| 39295 | ZV26400 | [V]Infertility general advice and counselling |  |  |
| 40072 | 4Z0..00 | Infertility studies |  |  |
| 69751 | ZV26z00 | [V]Unspecified infertility management |  |  |
| 93988 | 7E29200 | Fertiloscopy |  |  |
| 94448 | K5B1000 | Primary pituitary – hypothalamic infertility |  |  |
| 99535 | K5B1100 | Secondary pituitary – hypothalamic infertility |  |  |
| 101598 | K26y300 | Infertility due to radiation |  |  |
| 102589 | 8Cf..00 | Infertility care |  |  |
|  |  |  |  |  |

| 2.4 Specific fertility treatment codes | | | | |
| --- | --- | --- | --- | --- |
| Medcode | **Read code** | **Description** |  |  |
| 1810 | 8C8..00 | Treatment for infertility |  |  |
| 9983 | 8C8Z.00 | Treatment for infertility NOS |  |  |
| 8770 | SP0D100 | Hyperstimulation of ovaries |  |  |
| 1938 | 8C8Z.11 | In-vitro fertilisation |  |  |
| 8981 | ZV26800 | [V]Other assisted fertilization methods |  |  |
| 10238 | 8C84.11 | IVF |  |  |
| 11473 | 8C84.00 | In vitro fertilisation procedure |  |  |
| 30046 | ZV26700 | [V]In vitro fertilization |  |  |
| 52626 | 7M0h.00 | In vitro fertilisation (IVF) |  |  |
| 86010 | 7M0h211 | In vitro fertilisatn with intra-cytoplasmic sperm injection |  |  |
| 90936 | 7M0hz00 | In vitro fertilisation (IVF) NOS |  |  |
| 91845 | 7M0h511 | In vitro fertilisation with surrogacy |  |  |
| 91910 | 7M0h011 | In vitro fertilisation with donor sperm |  |  |
| 93810 | 7M0hy00 | Other specified in vitro fertilisation (IVF) |  |  |
| 94632 | 7M0h111 | In vitro fertilisation with donor eggs |  |  |
| 95677 | 7M0h411 | In vitro rtific with pre-implantation for genetic diagnosis |  |  |
| 97044 | 7M0h311 | In vitro fertilis intra-cytoplasmic sperm inj and donor egg |  |  |
| 98068 | Zvu2100 | [X]Other assisted fertilization methods |  |  |
| 102402 | 14e..00 | History of in-vitro fertilisation |  |  |
| 37828 | SP0D.00 | Complications associated with artificial fertilization |  |  |

| 2.5 Codes for pregnancy related to infertility | | | | |
| --- | --- | --- | --- | --- |
| Medcode | **Read code** | **Description** |  |  |
| 52685 | ZV23000 | [V]Pregnancy with history of infertility |  |  |
| 99069 | Kyu9G00 | [X]Female infertility of other origin |  |  |
| 37047 | 6242 | A/N care: H/O infertility |  |  |

| 2.6 Exposure: BNF codes, drug substance names and codes for drugs used in Ovulation Induction and ART  Note: Metformin and Tamoxifen used only in the presence of fertility-related codes, and with the exclusion of diabetes and breast cancer codes. | | | | |
| --- | --- | --- | --- | --- |
| BNF code | BNF Header | Drug substance name | gemscriptcode | prodcode |
| 6050100 | Hypothalamic And Anterior Pituitary Hormones And Anti-oestrogens | Clomifene citrate | 82240020 | 19128 |
|  |  |  | 57590020 | 22264 |
|  |  |  | 58900020 | 34862 |
|  |  |  | 61639020 | 37046 |
|  |  | Chorionic Gonadotrophin Human | 51234020 | 14520 |
|  |  |  | 54634020 | 18982 |
|  |  |  | 54635020 | 18981 |
|  |  |  | 54636020 | 10642 |
|  |  |  | 57574020 | 8763 |
|  |  |  | 57578020 | 4201 |
|  |  |  | 57582020 | 3129 |
|  |  |  | 61317020 | 10436 |
|  |  |  | 61321020 | 3519 |
|  |  |  | 61322020 | 15804 |
|  |  |  | 61326020 | 10702 |
|  |  |  | 61327020 | 8495 |
|  |  |  | 61330020 | 33422 |
|  |  |  | 74567020 | 2517 |
|  |  | Corticotropin | 61613020 | 1235 |
|  |  | Follitropin Alfa | 82407020 | 2518 |
|  |  |  | 82399020 | 12660 |
|  |  |  | 77520020 | 26623 |
|  |  |  | 86386020 | 32286 |
|  |  |  | 82408020 | 13662 |
|  |  |  | 82400020 | 14643 |
|  |  |  | 82409020 | 38436 |
|  |  |  | 82401020 | 45336 |
|  |  | Follitropin Beta | 83364020 | 3078 |
|  |  |  | 83365020 | 4378 |
|  |  |  | 83362020 | 12643 |
|  |  |  | 83366020 | 12649 |
|  |  |  | 85914020 | 15226 |
|  |  |  | 85921020 | 15249 |
|  |  |  | 83360020 | 20574 |
|  |  |  | 83361020 | 20575 |
|  |  |  | 85913020 | 12810 |
|  |  |  | 85571020 | 12870 |
|  |  |  | 85918020 | 12876 |
|  |  |  | 85920020 | 12906 |
|  |  | Gonadorelin hydrochloride | 53890020 | 25229 |
|  |  |  | 63050020 | 41438 |
|  |  |  | 63059020 | 22388 |
|  |  | Human Menopausic Gonadotrophin | 75071020 | 2512 |
|  |  | Menotrophin | 68962020 | 2513 |
|  |  |  | 57570020 | 3130 |
|  |  |  | 80814020 | 15954 |
|  |  |  | 84188020 | 15957 |
|  |  |  | 86026020 | 5438 |
|  |  | Urofollitropin | 75061020 | 2515 |
|  |  |  | 57566020 | 2760 |
|  |  |  | 75060020 | 3154 |
|  |  |  | 75919020 | 13490 |
|  |  |  | 69556020 | 3464 |
|  |  |  | 69557020 | 10441 |
|  |  |  | 6869020 | 51231 |
| 6050150 | Anti-oestrogens | Clomifene citrate | 61460020 | 1271 |
|  |  |  | 48707020 | 1775 |
|  |  |  | 63238020 | 38962 |
|  |  |  | 6868020 | 50064 |
| 6050151 | Anterior Pituitary Hormones - Corticotrophins | Urofollitropin | 92236020 | 39504 |
|  |  |  | 92234020 | 38094 |
|  |  |  | 92232020 | 39383 |
|  |  |  | 92238020 | 40562 |
| 6050152 | Anterior Pituitary Hormones -gonadotrophins | Choriogonadotropin alfa | 87924020 | 6595 |
|  |  |  | 87922020 | 32492 |
|  |  |  | 39238020 | 57051 |
|  |  | Chorionic gonadotrophin human | 57586020 | 2761 |
|  |  |  | 61320020 | 3465 |
|  |  |  | 51236020 | 6933 |
|  |  |  | 84299020 | 16028 |
|  |  |  | 51235020 | 21092 |
|  |  |  | 61325020 | 23889 |
|  |  | Follitropin alfa/Lutropin alfa | 80942020 | 15949 |
|  |  |  | 79209020 | 15981 |
|  |  |  | 87916020 | 19230 |
|  |  |  | 87920020 | 21579 |
|  |  |  | 87918020 | 23556 |
|  |  |  | 87912020 | 32485 |
|  |  |  | 87914020 | 32491 |
|  |  |  | 87910020 | 32494 |
|  |  |  | 79211020 | 32495 |
|  |  |  | 80869020 | 33279 |
|  |  |  | 14224020 | 52170 |
|  |  |  | 94124020 | 42711 |
|  |  | Follitropin beta | 85916020 | 4797 |
|  |  |  | 85917020 | 6316 |
|  |  |  | 85569020 | 12805 |
|  |  |  | 85570020 | 12832 |
|  |  |  | 89146020 | 14316 |
|  |  |  | 89228020 | 15299 |
|  |  |  | 89224020 | 26038 |
|  |  |  | 89222020 | 28179 |
|  |  |  | 89226020 | 33531 |
|  |  |  | 89144020 | 39627 |
| 6050153 | Human Menopausal Gonadotrophins | Menotrophin | 78437020 | 6125 |
|  |  |  | 91187020 | 7387 |
|  |  |  | 91189020 | 35966 |
|  |  |  | 80798020 | 36460 |
|  |  |  | 99934020 | 47031 |
|  |  |  | 46590020 | 58308 |
| 6070200 |  | Cetrorelix acetate | 86282020 | 30716 |
|  |  |  | 86283020 | 33446 |
|  |  | Ganirelix | 78657020 | 28178 |
| 06050100/06050154 | Hypothalamic And Anterior Pituitary Hormones And Anti-oestrogens/Hypothalamic Hormones | Gonadorelin hydrochloride | 52036020 | 37618 |
| 06050100/08030401 | Hypothalamic And Anterior Pituitary Hormones And Anti-oestrogens/Breast Cancer | Tamoxifen Citrate | 66868020 | 300 |
|  |  |  | 66869020 | 1416 |
|  |  |  | 58410020 | 3648 |
|  |  |  | 53266020 | 7346 |
|  |  |  | 50747020 | 7936 |
|  |  |  | 53270020 | 9075 |
|  |  |  | 63522020 | 11389 |
|  |  |  | 58407020 | 15038 |
|  |  |  | 74884020 | 21887 |
|  |  |  | 74883020 | 21888 |
|  |  |  | 79825020 | 22013 |
|  |  |  | 58413020 | 23972 |
|  |  |  | 66873020 | 24217 |
|  |  |  | 66874020 | 24238 |
|  |  |  | 79824020 | 26224 |
|  |  |  | 69491020 | 26227 |
|  |  |  | 69492020 | 26273 |
|  |  |  | 74885020 | 26306 |
|  |  |  | 65529020 | 26956 |
|  |  |  | 50665020 | 29607 |
|  |  |  | 57132020 | 30919 |
|  |  |  | 53481020 | 33470 |
|  |  |  | 53486020 | 33670 |
|  |  |  | 50664020 | 34010 |
|  |  |  | 60635020 | 34513 |
|  |  |  | 50675020 | 41590 |
|  |  |  | 57131020 | 41598 |
|  |  |  | 79826020 | 42098 |
|  |  |  | 59521020 | 45330 |
|  |  |  | 62641020 | 47841 |
|  |  |  | 53480020 | 55963 |
|  |  |  | 34520020 | 57312 |

**3. Costing methods for CPRD data**

3.1 Consultations:

Consultations were grouped by type (face-to-face surgery, visit, telephone) and staff role (GP, nurse). Administration activities (e.g referral letter, mails) by GP and nurses were viewed as a part of the consultation with no extra cost. We also observed activities conducted by administrative staffs and other staffs (e.g. Pharmacist, Acupuncturist) in the CPRD data. We consulted this with a colleague (Professor of Primary Care and a GP). It was suggested that activities conducted by administrative staffs are not consultations and are letters prepared from administrative staff that do not incur an additional cost; and the reason that we observed some “Other” roles for some consultations is that there are practices that offer some services which are not considered part of the primary care services, whereas other practices do not. As a result, activities with role of staff of “Administrative” & “Other” were not included in the analysis

Consultation categorising:

| **Consultation name** | **Consultation category** |
| --- | --- |
| Clinic | Face-to-face-surgery |
| Night visit, Deputising service | Visit |
| Follow-up/routine visit | Visit |
| Night visit, Local rota | Visit |
| Mail from patient | Admin |
| Night visit , practice | Visit |
| Out of hours, Practice | Visit |
| Out of hours, Non Practice | Visit |
| Surgery consultation | Face-to-face-surgery |
| Telephone call from a patient | Telephone |
| Acute visit | Visit |
| Discharge details | Admin |
| Letter from Outpatients | Admin |
| Repeat Issue | Admin |
| Other | Admin |
| Results recording | Admin |
| Mail to patient | Admin |
| Emergency Consultation | Face-to-face-surgery |
| Administration | Admin |
| Casualty Attendance | Not considered as primary care |
| Telephone call to a patient | Telephone |
| Third Party Consultation | Admin |
| Hospital Admission | Not considered as primary care |
| Children's Home Visit | Visit |
| Day Case Report | Admin |
| GOS18 Report | Admin |
| Home Visit | Visit |
| Hotel Visit | Visit |
| NHS Direct Report | Admin |
| Nursing Home Visit | Visit |
| Residential Home Visit | Visit |
| Twilight Visit | Visit |
| Triage | Telephone |
| Walk-in Centre | Face-to-face-surgery |
| Co-op Telephone advice | Telephone |
| Co-op Surgery Consultation | Face-to-face-surgery |
| Co-op Home Visit | Visit |
| Minor Injury Service | Not considered as primary care |
| Medicine Management | Admin |
| Community Clinic | Not considered as primary care |
| Data Transferred from other system | Admin |
| Health Visitor Report | Admin |
| Hospital Inpatient Report | Admin |
| Night Visit | Visit |
| Radiology Result | Admin |
| Referral Letter | Admin |
| Telephone Consultation | Telephone |
| Template Entry | Admin |
| GP to GP communication transaction | Admin |
| Non-consultation medication data | Admin |
| Non-consultation data | Admin |

Staff role categorizing:

| **Role Of Staff** | **Role category** |
| --- | --- |
| Senior Partner | GP |
| Partner | GP |
| Assistant | GP |
| Associate | GP |
| Non-commercial local rota of less than 10 GPs | GP |
| Commercial Deputising service | GP |
| Locum | GP |
| GP Registrar | GP |
| Consultant | Other |
| Sole Practitioner | GP |
| Practice Nurse | Nurse |
| Health Visitor | Other |
| Community Nurse | Nurse |
| Midwife | Other |
| Community Psychiatric Nurse | Other |
| Social Worker | Other |
| Pharmacist | Other |
| Dispenser | Other |
| Non-qualified Dispenser | Other |
| Practice Manager | Admin |
| Fund Manager | Admin |
| Business Manager | Admin |
| Administrator | Admin |
| Secretary | Admin |
| Receptionist | Admin |
| Physiotherapist | Other |
| Chiropodist | Other |
| Dentist | Other |
| Dietician | Other |
| Counsellor | Other |
| Osteopath | Other |
| Maintenance staff | Admin |
| Other Health Care Professional | Other |
| Hospital Nurse | Other |
| Community Medical Officer | Other |
| School Nurse | Other |
| Health Education Officer | Other |
| Contact Tracing Nurse | Other |
| Stomatherapist | Other |
| Computer Manager | Admin |
| Interpreter/Link Worker | Other |
| Chiropractor | Other |
| Acupuncturist | Other |
| Homeopath | Other |
| Mental Handicap Nurse | Other |
| Carer | Other |
| Salaried Partner | GP |
| Occupational Therapist | Other |
| Speech Therapist | Other |
| GP Retainer | GP |
| Phlebotomist | Other |
| Other Medical & Dental | Other |
| Other Students | Other |
| Other Nursing & Midwifery | Other |
| Other Allied Health Professionals | Other |
| Other Professional Scientific & Technical | Other |
| Other Healthcare Scientists | Other |
| Other Additional Clinical Services | Other |
| Other Admin & Clerical | Admin |
| Clinical Practitioner Access Role | Other |
| Nurse Access Role | Other |
| Nurse Manager Access Role | Other |
| Health Professional Access Role | Other |
| Healthcare Student Access Role | Other |
| Biomedical Scientist Access Role | Other |
| Clinical Coder Access Role | Admin |
| Optometrist | Other |
| Radiographer | Other |

Since 2016, only GP face-to-face consultation duration was reported in the Unit Costs of Health and Social Care report and there was no duration reported for other categories. So we have used the 2015 report to collect unit cost --

|  | GP | Nurse |
| --- | --- | --- |
| Face to face surgery | £44 (11.7min) | £14  (15.5min) |
| Visit | £89  (11.4+12min) | £34  (25+12min) |
| Telephone | £27  (7.1min) | £4  (6min) |

The above unit cost for consultations were then inflated to 2018/19 ones using the New Health Services Index using CPI (Health) published in 2019 Unit Costs of Health and Social Care report.

3.2 Test:

We grouped tests into broad categories as attached unit cost collected from the 2018/19 NHS reference cost schedule. Weighted average was used when appropriate.

| Test category | Unit cost |
| --- | --- |
| Clinical biochemistry | £1 |
| Haematology | £3 |
| Immunology | £7 |
| Microbiology | £8 |
| Cytology | £7 |
| Histology | £40 |
| X-ray | £31 |
| Endoscopy | £490 |
| MRI | £180 |
| CT | £110 |
| Fluoroscopy | £150 |
| Ultrasound | £56 |
| Other diagnostic imaging | £97 |
| Nuclear medicine | £359 |
| Angiogram/venography | £43 |
| ECG | £49 |
| EEG | £330 |
| Dexa | £73 |
| Genetics screening | £29 |
| Lung volumn study | £117 |
| Nerve conduction studies | £330 |

4.3 Referral:

Referral records were used as a proxy of patients’ first visit to outpatient clinics in the study. Emergency admissions and attendance at Accident & Emergency were not included in the analysis. An NHS specialty code was available for each referral record, which was mapped to the NHS specialty and corresponding unit cost in the 2018/19 NHS reference cost schedule.

3.4 Prescription:

A BNF code was available for each prescription in the CPRD data, which can be mapped to the net ingredient cost reported in the *Prescription Cost Analysis (PCA): England*. The first 2 digits of the BNF codes represent BNF chapters (e.g. *01 Gastro-intestinal system*). The 3&4 digits of the BNF codes represent BNF section (e.g. *0101 Dyspep&Gastro-Oesophageal Reflux Disease*). The 5&6 digits of the BNF codes represent BNF paragraph (e.g. *010101 Antacids and simethicone*). Around 89% of prescription records is the study were matched to unit cost on BNF paragraph level, 9% were matched on section level, the rest were matched on chapter level.

In the 2019 PCA unit costs were not available on BNF paragraph level. As results we have used unit costs reported in the 2018 PCA and inflated them into 2019 values using the New Health Services Index using CPI (Health) published in 2019 Unit Costs of Health and Social Care report.

**4. Inverse probability weights (IPW) methods**

1) Within each of the fertility groups, we estimated Cox regression with “being censored” (patients transfer out of the contributing GP practice, emigration) as the outcome and mum's delivery age, socioeconomic deprivation, year, geographic region of GP practice as covariates

2) Based on the Cox regressions, we estimated the “probability of not being censored” at the end of each time interval at individual level taking consideration of the mother and baby characteristics list in step 1

3) IPW were calculated as the reciprocal of the probabilities estimated in step 2

4) IPW calculated in step 3 were used as sampling weights in linear regressions in each time interval to compare the cost in different fertility groups
